# FUT8 Is a Critical Driver of Prostate Tumour Growth and Can Be Targeted Using Fucosylation Inhibitors

**DOI:** 10.1002/cam4.70959

**Published:** 2025-05-19

**Authors:** Kayla Bastian, Margarita Orozco‐Moreno, Huw Thomas, Kirsty Hodgson, Eline A. Visser, Emiel Rossing, Johan F. A. Pijnenborg, Nienke Eerden, Laura Wilson, Hasvini Saravannan, Oliver Hanley, Grace Grimsley, Fiona Frame, Ziqian Peng, Bridget Knight, Paul McCullagh, John McGrath, Malcolm Crundwell, Lorna Harries, Norman J. Maitland, Rakesh Heer, Ning Wang, Ethan D. Goddard‐Borger, Ramon Hurtado Guerrero, Thomas J. Boltje, Richard R. Drake, Emma Scott, David J. Elliott, Jennifer Munkley

**Affiliations:** ^1^ Newcastle University Centre for Cancer Newcastle University Institute of Biosciences Newcastle UK; ^2^ Newcastle University Centre for Cancer, Translational and Clinical Research Institute, Paul O'gorman Building Newcastle University Newcastle upon Tyne UK; ^3^ Synthetic Organic Chemistry, Institute for Molecules and Materials Radboud University Nijmegen the Netherlands; ^4^ GlycoTherapeutics B.V Nijmegen the Netherlands; ^5^ Department of Cell and Molecular Pharmacology Medical University of South Carolina Charleston South Carolina USA; ^6^ Cancer Research Unit, Department of Biology University of York North Yorkshire UK; ^7^ NIHR Exeter Clinical Research Facility Royal Devon and Exeter NHS Foundation Trust Exeter UK; ^8^ Department of Pathology Royal Devon and Exeter NHS Foundation Trust Exeter UK; ^9^ Exeter Surgical Health Services Research Unit Royal Devon and Exeter NHS Foundation Trust Exeter UK; ^10^ Institute of Biomedical and Clinical Sciences, Medical School, College of Medicine and Health University of Exeter Exeter UK; ^11^ The Mellanby Centre for Musculoskeletal Research, Division of Clinical Medicine The University of Sheffield Sheffield UK; ^12^ Leicester Cancer Research Centre, Department of Genetics, Genomics, and Cancer Sciences University of Leicester Leicester UK; ^13^ The Walter and Eliza Hall Institute of Medical Research Parkville Victoria Australia; ^14^ Department of Medical Biology University of Melbourne Parkville Victoria Australia; ^15^ University of Zaragoza Zaragoza Spain; ^16^ Copenhagen Center for Glycomics, Department of Cellular and Molecular Medicine University of Copenhagen Copenhagen Denmark

**Keywords:** core fucosylation, fucosylation inhibitors, fucosyltransferase 8 (FUT8), glycans, prostate cancer, therapeutics, tumour growth

## Abstract

**Background:**

An unmet clinical need requires the discovery of new treatments for men facing advanced prostate cancer. Aberrant glycosylation is a universal feature of cancer cells and plays a key role in tumour growth, immune evasion and metastasis. Alterations in tumour glycosylation are closely associated with prostate cancer progression, making glycans promising therapeutic targets. Fucosyltransferase 8 (FUT8) drives core fucosylation by adding α1,6‐fucose to the innermost GlcNAc residue on *N*‐glycans. While FUT8 is recognised as a crucial factor in cancer progression, its role in prostate cancer remains poorly understood.

**Methods & Results:**

Here, we demonstrate using multiple independent clinical cohorts that FUT8 is upregulated in high grade and metastatic prostate tumours, and in the blood of prostate cancer patients with aggressive disease. Using novel tools, including PhosL lectin immunofluorescence and *N*‐glycan MALDI mass spectrometry imaging (MALDI‐MSI), we find FUT8 underpins the biosynthesis of malignant core fucosylated *N*‐glycans in prostate cancer cells and using both in vitro and in vivo models, we find FUT8 promotes prostate tumour growth, cell motility and invasion. Mechanistically we show FUT8 regulates the expression of genes and signalling pathways linked to prostate cancer progression. Furthermore, we find that fucosylation inhibitors can inhibit the activity of FUT8 in prostate cancer to suppress the growth of prostate tumours.

**Conclusions:**

Our study cements FUT8‐mediated core fucosylation as an important driver of prostate cancer progression and suggests targeting FUT8 activity for prostate cancer therapy as an exciting area to explore.

## 1 Introduction

Prostate cancer is the second most common cancer in men worldwide resulting in 375,000 deaths annually [1, 2]. Localised prostate cancer is largely curable and has a 5‐year survival rate of more than 99%, but for advanced prostate cancer only 32% of patients will still be alive after 5 years [3]. The growth of prostate tumours is driven by androgen receptor (AR) signalling and initial therapeutic options for advanced prostate cancer are hormone‐based therapies such as anti‐androgens [4, 5, 6]. Androgen deprivation therapy (ADT) typically leads to tumour shrinkage, but tumours inevitably relapse into the lethal form of the disease, termed castration‐resistant prostate cancer (CRPC), where the acquisition of resistance mechanisms mean tumours persist despite low androgen conditions [7, 8]. Patients with CRPC can be managed with second generation AR inhibitors (enzalutamide, abiraterone, apalutamide, or darolutamide), chemotherapy, immunotherapy, poly‐ADP ribose polymerase (PARP) inhibitors or radium‐233 for bone metastases [3, 9, 10, 11, 12]. However, resistance to these treatments is very common and once CRPC occurs the median survival rate for patients is only 9–30 months, meaning there is an urgent unmet clinical need to develop new therapeutic interventions [13, 14].

Altered glycosylation is a hallmark of cancer that is closely linked to a malignant phenotype [15, 16, 17]. Cancer‐associated glycans can directly impact key processes supporting tumour growth, metastasis and immune evasion and are an area of innovation in the search for new cancer therapies [18, 19, 20]. A widely occurring cancer‐associated change in glycosylation is altered fucosylation [17]. Fucosylation is a type of glycosylation where fucose residues are attached to glycans and can be divided into either terminal or core fucosylation. A family of 13 fucosyltransferase (FUT) enzymes catalyse fucosylation [21, 22, 23, 24]. Core fucosylation is catalysed by α1,6 fucosyltransferase 8 (FUT8) which transfers fucose to the innermost GlcNAc of *N*‐linked glycoproteins by an α1,6 linkage [22, 25, 26]. FUT8 is the only FUT enzyme responsible for core fucosylation, and as most other fucosyltransferases are functionally redundant this makes FUT8 and core fucosylation unique [27, 28, 29, 30, 31]. The therapeutics field has long benefited from ablating the *FUT8* gene in antibody producing cells, and it is well established that this can enhance the effector functions of antibodies [32]. FUT8‐mediated core fucosylation also plays an important role in cancer biology, and upregulation of FUT8 has been identified in numerous cancer types including lung [33, 34, 35], liver [36], colorectal [37, 38, 39], thyroid [40], melanoma [41], pancreatic [42], ovarian [43, 44], breast [45, 46, 47, 48] and prostate cancer [49, 50]. In lung cancer, FUT8 can globally modify surface antigens, receptors and adhesion molecules and knockdown of FUT8 in aggressive cell lines inhibits in vivo tumour growth and metastasis [33]. A systems biology approach recently identified core fucosylation as a crucial factor in the aggressive behaviour of melanoma cells and showed FUT8 is a key driver of melanoma metastasis [41]. Similarly, in breast cancer, a network of core fucosylated glycoproteins with functional roles in breast cancer progression have been identified and linked to metastasis [45, 47]. For prostate cancer, core fucosylation of prostate specific antigen (PSA) has been widely investigated as a biomarker for aggressive disease [51, 52, 53, 54, 55] and serum fucosylated haptoglobin is known to be upregulated in high grade disease [56].

FUT8 has previously been reported as upregulated in high grade and metastatic prostate cancer and is linked increased cell motility and the development of CRPC [49, 50], however these studies were based on cell lines and small numbers of clinical samples and did not specifically investigate the in vivo functional role of FUT8. Furthermore, FUT8 is yet to be investigated as a potentially important clinical target in prostate cancer. Here we monitor FUT8 levels in > 1500 clinical samples across multiple patient cohorts and verify that FUT8 is upregulated in high grade and metastatic prostate tumours and in the blood of prostate cancer patients with aggressive prostate disease. Using novel tools, including PhosL lectin immunofluorescence and *N*‐glycan MALDI mass spectrometry imaging (MALDI‐MSI), we find FUT8 regulates the expression of malignant core fucosylated *N*‐glycans in prostate cancer cells, and using both in vitro and in vivo models, we show FUT8 can promote prostate tumour growth and increase cell migration and invasion. Using RNA‐sequencing, we reveal FUT8 controls the expression of genes and proteins linked to disease progression. Furthermore, we reveal that the action of FUT8 can be targeted in vivo using fucosyltransferase inhibitors. Our study identifies FUT8‐mediated core fucosylation as an important player in aggressive prostate cancer and highlights the targeting of FUT8 activity as a promising new strategy for prostate cancer therapy.

## 2 Methods

### 2.1 Cell Culture

Cell culture of cells was as described previously [57]. PC3 (CRL‐1435), DU145 (HTB‐81) and CW‐22Rv1 (CRL‐250) cells were obtained from ATCC (CRL‐1435 and CRL‐2505). All cells were cultured at 37°C, 5% CO_2_ in a humidified incubator, and passaged with trypsin every 3–4 days. Stable cell lines were created using lentiviral transduction. For FUT8 knockdown, shRNA lentiviral particles were purchased from Santa Cruz (FUT8 shRNA sc‐45757‐V and Control shRNA sc‐108080). Transductions were carried out according to the manufacturer's instructions using MOI = 5. For FUT8 overexpression, Lentifect purified lentiviral particles were purchased from Tebu‐Bio (FUT8 LPP‐A1604‐Lv242‐050 and negative control 217LPP‐NEG‐Lv242‐025‐C). Transductions were carried out according to the manufacturer's instructions using MOI = 5. Cell lines were authenticated using DNA STR analysis and tested every 3 months for mycoplasma contamination.

### 2.2 Real‐Time PCR

Total RNA was isolated from cultured cell lines using a RNeasy Mini Kit (Qiagen 205,411) and treated with DNase 1 (Ambion, AM2222). cDNA synthesis was performed using a Superscript VILO cDNA synthesis kit (Invitrogen, 15,596–026) according to the manufacturer's instructions. Real‐time quantitative PCR (RT‐qPCR) was carried out as previously described [57, 58]. Briefly, cDNA was tested in triplicate using SYBR Green PCR Master Mix (Invitrogen, 4,309,155) using the QuantStudio 7 Flex Real‐Time PCR System (Life Technologies). All samples were normalised using the average of three reference genes (actin, tubulin and GAPDH). Primer sequences are provided in Table S1.

### 2.3 Lectin Immunofluorescence

Lectin immunofluorescence was as described previously [59]. Cells were cultured in Lab‐TekII Chamber Slides (Thermo Scientific, 154,453) in complete media. After 72 h, cells were washed with PBS before permeabilization and fixation with ice‐cold absolute methanol for 10 min at −20°C. Next, slides were washed with PBS and blocked with 1X Carbo‐Free Blocking Solution (1X CFB) (Vector Laboratories, SP‐5040‐125) for 1 h at room temperature. Slides were incubated for 3 h at room temperature with 1:1000 biotinylated PhoSL (a novel lectin from the mushroom *Pholiota squarrosa* which specifically recognises core‐fucose [60], kindly gifted to us by Professor Ethan Goddard). Subsequently, cells were stained with Streptavidin AZDye 647 (Abcam, ab272190) for 1 h at room temperature in dark conditions. Finally, slides were washed with PBS and stained with Hoechst (Thermo Scientific, 62,249) for 15 min at room temperature. Cells were mounted using ProLong Gold Antifade reagent (Thermo Fisher, P36930). Images were acquired and processed with the ZEISS Axio Imager 3.

### 2.4 Immunocytochemistry

Immunocytochemistry assays were performed as described previously [61]. Briefly, cells were cultured for 72 h in complete media in Lab‐TekII Chamber Slides (Thermo Scientific, 154,453). Cells were then washed with PBS before permeabilization and fixation with ice‐cold absolute methanol at −20°C for 10 min. Slides were washed with PBS and blocked with 10% goat serum (Abcam, ab7481) for 1 h at room temperature. Slides were incubated overnight at 4°C with FUT8 rabbit polyclonal antibody (Sigma, HPA043410), PTGES3 mouse monoclonal antibody (Proteintech, 67,736‐1‐Ig), IGFBP5 rabbit polyclonal antibody (Proteintech, 55,205‐1‐AP) or IL1B rabbit polyclonal antibody (Proteintech, 16,806‐1‐AP) followed by 1 h incubation with appropriate secondary antibodies. Finally, slides were washed with PBS and stained with Hoechst (Thermo Scientific, 62,249) for 15 min at room temperature. Images were acquired and processed with the ZEISS Axio Imager 5.

### 2.5 Immunohistochemistry

For immunohistochemistry analysis of FUT8 protein levels in prostate cancer tissue microarrays (TMAs), antigen retrieval was performed by pressure cooking for 90 s in 10 mM citrate pH 6.0 (Sigma‐Aldrich, C9999) followed by staining with FUT8 antibody (Sigma HPA043410, 1:100). Nuclei were counterstained with haematoxylin (Sigma‐Aldrich, 51,275). Slides were scanned using an Aperio CS2 (Leica biosystems) and the levels of FUT8 were assessed using the cytoplasmic v2 algorithm. In some cases, an epithelia mask was included to identify the epithelia in the sample before running the cytoplasmic v2 algorithm. The FUT8 antibody was validated using formalin‐fixed paraffin‐embedded (FFPE) cell pellets with knockdown of FUT8 (Figure S2).

### 2.6 ELISA Assays

Human FUT8 sandwich ELISA kits were purchased from Cambridge Bioscience (RayBioTech, ELH‐FUT8). Samples and standards were assayed in duplicate according to the manufacturer's protocol. Conditioned media samples were prepared as described previously [58, 61].

### 2.7 Clinical Samples

#### 2.7.1 RNA‐Sequencing Data

*FUT8* mRNA levels in the TCGA Firehouse Legacy cohort [62] and the *Cancer Cell* 2018 cohort [63] were analysed using cBioportal [64, 65] as described previously [58] (Figures S1A,B).

#### 2.7.2 RNA From Clinical Tissue

**FIGURE 1:**
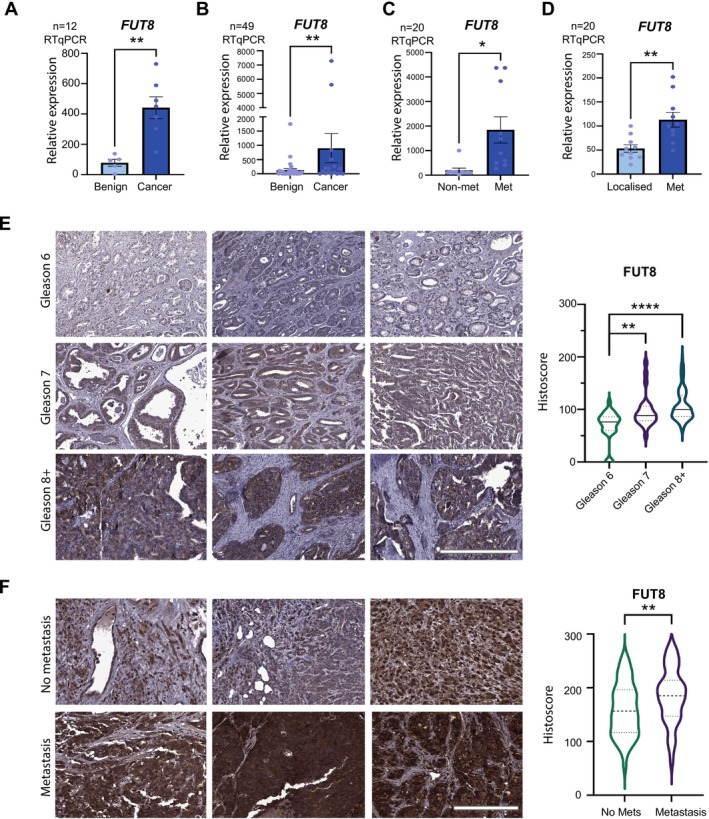
FUT8 is upregulated in high grade and metastatic prostate tumours. (A–D) *FUT8* gene expression levels were detected in clinical samples using real‐time quantitative PCR (RT‐qPCR). (A) *FUT8* mRNA levels were significantly higher in prostate cancer relative to benign prostate hyperplasia (BPH) (*n* = 12, unpaired t‐test, *p* < 0.01, **). (B) *FUT8* mRNA was monitored in a cohort of 33 BPH and 16 prostate cancer samples using real‐time PCR. *FUT8* levels were higher in prostate cancer relative to BPH (*n* = 49, unpaired t‐test, *p* < 0.01, **). (C) Higher *FUT8* expression was also detected in a sub‐group of prostate tumours with ‘metastatic’ biology compared to tumours with a ‘non‐metastatic’ phenotype [67] (*n* = 20, unpaired t‐test, *p* < 0.05, *). (D) *FUT8* gene expression levels were also significantly increased in metastatic prostate cancer relative to localised disease (*n* = 20, unpaired *t*‐test, *p* < 0.01, **). (E) Immunohistochemistry (IHC) analysis of FUT8 protein levels in a previously published tissue microarray (TMA) [58, 61]. The levels of FUT8 were significantly higher in both Gleason grade 7 tumours (including both 3 + 4 and 4 + 3 tumours) and Gleason grade 8–10 tumours compared to Gleason grade 6 tumours (*n* = 80, unpaired t test, *p* = 0.0029 ** and *p* < 0.0001, ****). Scale bar is 300 μm. (F) Immunohistochemistry analysis of a previously published 125 case TMA [68, 69] to compare FUT8 levels in localised prostate cancer tumours and in prostate cancer tissues presenting with metastasis (all biopsy samples were taken from the primary site). FUT8 levels are significantly higher in metastatic tumours compared to localised tumours (*n* = 125, unpaired t test, *p* = 0.0084, **). Scale bar is 200 μm.

*FUT8* mRNA levels were monitored using real‐time qPCR in four previously published patient cohorts [57, 58, 66, 67] (Figure 1A–D).

#### 2.7.3 Prostate Cancer Tissue Microarrays (TMAs)

FUT8 protein levels were monitored using immunohistochemistry in two previously published prostate cancer TMAs, including a 96 case TMA (US Biomax, PR1921b) [58, 61] (Figure 1E) and an intermediate density TMA comprising 125 cases of advanced prostate cancer presenting with either localised prostate cancer or prostate patients presenting with metastasis (all biopsy samples were taken from the primary site) [68, 69] (Figure 1F).

#### 2.7.4 Blood Samples

**FIGURE 2:**
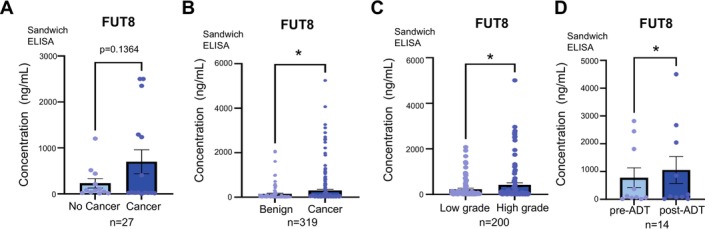
FUT8 protein levels are increased in the blood of patients with aggressive prostate cancer. (A‐D) Detection of FUT8 protein in blood samples from patients with prostate cancer using sandwich ELISA assays. (A) FUT8 levels were 3.02‐fold higher in plasma samples from patients with prostate cancer compared to patients given a no‐cancer diagnosis (*n* = 27, unpaired t test, *p* = 0.109). (B) The levels of FUT8 protein were 2.09‐fold higher in plasma samples from men with prostate cancer compared to men diagnosed with BPH (*n* = 319, unpaired t test, *p* = 0.0457, *). (C) FUT8 levels were 1.86‐fold increased in serum samples from patients with high grade prostate cancer (Gleason grade 8–9) compared to patients with low grade prostate cancer (Gleason grade 6–7) (*n* = 200, unpaired t test, *p* < 0.0218, *). (D) Analysis of FUT8 levels in matched serum samples from 7 men with prostate cancer taken before and after ADT. FUT8 serum levels significantly increase after ADT (*n* = 14, paired t test, *p* = 0.047, *).

The plasma samples tested in Figure 2A were collected by the Exeter Clinical Research Facility tissue bank (Ref: STB20) during standard routine National Health Service (NHS) clinical practice and spun (at least 30 min after collection) at 4500 × g for 10 min. The separated plasma was removed, aliquoted, and stored at −80 °C. Written informed consent for the use of biological samples was provided by all patients. The patient plasma samples tested in Figure 2B were collected with ethical permission from Castle Hill Hospital (Cottingham, Hull) (ethics number: 07/H1304/121) and prepared using Histopaque (Sigma‐Aldrich, 1077) as per the manufacturer's instructions (samples were spun at 600 × g for 15 min at room temperature). Use of patient tissue was approved by the local research ethics committees. Patients gave informed consent, and all patient samples were anonymised. The serum samples analysed in Figure 2C,D were kindly provided by Dr. Colm Morrissey (University of Washington) via the Prostate Cancer Biorepository Network (PCBN). The serum samples in Figure 2C were taken from patients who had prostatectomies. For Figure 2D, the pre‐ADT samples were taken from prostate cancer patients undergoing radical prostatectomies prior to any hormonal therapy. Matched post‐ADT samples were taken from the same patients 1–4 months after beginning either Lupron or LH therapy. Our study was peer reviewed and approved by PCBN. Samples were prepared and stored using standard protocols and all patients gave informed consent.

### 2.8 *N*‐Glycan MALDI‐MSI

FFPE tissue was prepared for *N*‐glycan Matrix‐assisted laser desorption/ionisation mass spectrometry imaging (MALDI‐MSI) using previously published protocols for antigen retrieval, enzyme and matrix applications by solvent sprayer (M5, HTX Imaging, Durham, NC), and analysis on a timsTOF fleX QTOF mass spectrometer (Bruker Corp, Germany) [70, 71, 72]. A modification to the standard protocol was spraying a combined mixture of PNGaseF PRIME (100 mg/tissue) and Endoglycosidase F3 PRIME (10 mg/tissue, Endo F3) (N‐Zyme Scientifics, Doylestown, PA) to release *N*‐glycans. Endo F3 specifically cleaves core fucosylated *N*‐glycans, leaving a GlcNAc‐Fuc still attached to the protein, and a − 349 m/z glycan product [73]. Spectra and tissue images were annotated in SCiLS Lab software (v. 2024a) by matching peaks against an in‐house *N*‐glycan database [70, 72].

### 2.9 Lectin Flow Cytometry

Cells were cultured for 72 h in complete media containing DMSO (vehicle control) or the indicated concentrations of Fucotrim I. Cells were washed with PBS and harvested with trypsin and centrifugation (500 × *g*, 5 min at room temperature). The cells were washed twice with 1X Carbo‐Free Blocking Solution (1X CFB) (Vector labs, SP‐5040‐125) then resuspended in 100 μL of 1:2000 FITC‐conjugated AAL lectin (Vector Labs, FL‐1391‐1) or LCA lectin (Vector Labs, FL‐1041‐5) in 1X CFB and incubated for 30 min at 4°C. Cells were washed with PBS twice before being resuspended in 500 μL PBS with 1 μg/mL propidium iodide. 10,000 events per sample were acquired on a BD LSRFortessa Cell Analyser (BD Biosciences). Data was analysed using the FCS Express Flow Cytometry Analysis Software (the plots shown are representative of three biological repeats).

### 2.10 Proliferation Assays

WST1 and colony formation assays were performed as described previously [74]. For fucosylation inhibitor experiments, cells were pre‐treated with respective inhibitor/concentration for 3 days followed by 24‐h serum‐starvation.

### 2.11 Migration Assays

Cells were seeded into a 24‐well plate at a concentration of 2.5 × 10^5^ cells/well and cultured until a confluent monolayer was observed. Then, media was removed from each well and the monolayer was scratched horizontally across the middle section using a P1000 pipette tip. Residues from the scratch were removed by washing with PBS before adding fresh media (10% FBS) to the wells. Scratches were imaged using a light microscope with a 4X objective magnification at time 0 and every 8 h, until a maximum incubation time of 72 h. The area of scratch was defined and quantified using ImageJ software (v 1.53 s).

### 2.12 Invasion Assays

Assays were conducted in collagen‐coated Oris Pro 384‐well microplates (Platypus Technologies, PRO384CMACC5) as per the manufacturer's instructions. Cells were seeded in triplicate at 10 × 10^5^ cells/well and incubated for 2 h. Post migratory images were taken at 48 h. The area of the detection zone was measured both pre‐ and post‐invasion using ImageJ and the average percent closure was calculated.

### 2.13 Mouse Models

#### 2.13.1 PC3 Tumour Xenografts

3 × 10^6^ PC3 cells with FUT8 knockdown were implanted into the subcutaneous space of the right flank of 8‐week old Naval Medical Research Institute (NMRI) nude mice (*n* = 6 mice/group). The mice were randomised into control or treatment groups before cancer cell inoculation. Cells were injected in a volume of 50 μL of cell culture media and Matrigel in a 1:1 mixture. Animals were weighed and tumour volumes were monitored by calliper measurement three times a week by an unblinded researcher until the first animal met a humane endpoint (defined as tumour volume reaching 1000 mm^3^).

#### 2.13.2 CWR22RV1 Tumour Xenografts

Male CD‐1 nude mice (Charles Rivers) were inoculated at 7 weeks of age with 1 × 10^7^ CWR22RV1 cells with FUT8 overexpression by unilateral subcutaneous injection into the right flank (*n* = 10 mice/group). The mice were randomised into control or treatment groups before cancer cell inoculation. Cells were injected in a volume of 100 μL cell culture media and Matrigel in a 1:1 mixture. Animals were weighed and tumour volumes were monitored by calliper measurement three times a week by a blinded researcher until the first animal met a humane endpoint (defined as tumour volume reaching 1000 mm^3^). Tumours with ulceration were excluded from the analysis.

#### 2.13.3 SGN‐2FF Study

CD‐1 nude mice (Charles Rivers) were randomised to start treatment with either 150 mg/kg fucosylation inhibitor SGN‐2FF (Cambridge Bioscience, HY‐107366) or water via oral gavage daily 7 days prior to implantations (*n* = 10 mice/group). On day 7, CWR22Rv1 cells were subcutaneously injected into the right flank of 7‐week‐old CD‐1 nude mice (1.0 × 10^7^ cells in 50 μL Matrigel/media). Daily treatment by oral gavage for both groups continued for the duration of the study. Tumour size was measured up to 5 times weekly using callipers.

For subcutaneous xenograft models tumours were removed from the flank and prepared for histological analysis. Tumours were fixed in 10% neutral buffered formalin (Sigma HT501128‐4 L) for 24 h. After 24 h, tissue was washed in 70% ethanol to remove the residual formalin and to stop fixation before storing in fresh 70% ethanol for up to one month prior to processing. All animal experiments were approved by the Newcastle Ethical Review Committee and performed under a UK Home Office licence (PPL: PP5794374, Huw Thomas and PIL: I65375803, Kayla Bastian). All mice once obtained were housed with unrestricted access to food and water and maintained on a constant 12 h light–dark cycle.

### 2.14 RNA‐Sequencing

RNA sequencing data can be accessed on the GEO repository (submission GSE280132). RNA was extracted from cell lines transduced with negative‐control lentiviral particles or with stable FUT8 overexpression/knockdown particles with 3 biological repeats per experimental condition. Samples were prepared as described previously [74]. Overexpression samples and their respective controls were sequenced using an Illumina NextSeq 550, giving approximately 18 million single reads per sample. Knockdown samples and their respective controls were sequenced using an Illumina NovaSeq 6000 instrument, giving approximately 21 million single reads per sample. All data analyses were performed in Galaxy version 22.01 [75]. Quality control was performed with FastQC (http://www.bioinformatics.babraham.ac.uk/projects/fastqc/) and reads were trimmed with Cutadapt [76]. Reads were mapped to hg38 using HISAT2 [77] and quantified with featureCounts [78]. Differential gene expression analysis was performed using limma‐voom [79] and a volcano plot was generated with ggplot2 [80]. Gene ontology (GO) analysis was performed with goseq [81] applying a significance threshold of adjusted *p*‐value < 0.05 for differentially expressed genes. Gene Set Enrichment Analysis (GSEA) was performed with the package EGSEA [82]. Normalised count matrix values were used to create a heatmap with gplots [83].

### 2.15 Statistical Analysis

Statistics were performed using the GraphPad Prism software (version 9.4.1). Data are presented as the mean of three independent samples ± standard error of the mean (SEM). Statistical significance is indicated as *p < 0.05, **p < 0.01, ***p < 0.001 and ****p < 0.0001.

## 3 Results

### 3.1 The Fucosyltransferase Enzyme FUT8 Is Upregulated in High Grade and Metastatic Prostate Tumours

FUT8 has previously been identified as upregulated in prostate cancer tumours and linked with the development of high‐grade disease [49]. However, this study relied on cell lines and a relatively small number of clinical samples. Here, we monitor FUT8 expression at both the gene and protein level in 8 independent cohorts (comprising > 1500 clinical samples) and confirm upregulation of FUT8 in aggressive high grade prostate tumour tissue. Analysis of RNA sequencing data from The Cancer Genome Atlas Prostate Adenocarcinoma (TCGA PRAD) cohort [62] revealed *FUT8* levels are significantly higher in Gleason grade 7 and 8+ tumours, compared to Gleason grade 6 tumours (*n* = 595, *p* < 0.001, *p* < 0.01) (Figure S1A). Similarly, in the *Cancer Cell* 2018 cohort [63], *FUT8* levels were significantly higher in Gleason 8+ tumours compared to Gleason 6/7 prostate tumours (*n* = 118, *p* < 0.05) (Figure S1B). Previous studies have suggested that *FUT8* can be repressed by androgens [84, 85], and consistent with this, we detected an increase in *FUT8* levels in clinical samples from patients treated with ADT and a decrease in FUT8 levels in prostate cancer cells stimulated with androgens (Figure S1C–G). Real‐time quantitative PCR detected upregulation of the *FUT8* gene in prostate cancer relative to benign prostate hyperplasia (BPH) gland (*n* = 12, *p* < 0.01) (Figure 1A) which was further validated in a larger independent patient cohort (*n* = 49, *p* < 0.01) (Figure 1B). In additional cohorts of patients with prostate cancer, *FUT8* mRNA was upregulated in prostate cancers with a ‘metastatic’ signature compared to tumours with ‘non‐metastatic’ biology [67] (n20, *p* < 0.05) (Figure 1C) and in metastatic prostate tumours compared to localised disease (*n* = 20, *p* < 0.05) (Figure 1D). Next, to test if FUT8 is also upregulated at the protein level in high grade prostate tumours, we used immunohistochemistry (IHC) to monitor FUT8 protein levels in two previously published prostate cancer tissue microarrays (TMAs) [58, 61, 68, 69]. We confirmed the specificity of our FUT8 immunohistochemistry via detection of protein depletion in Formalin Fixed Paraffin embedded (FFPE) cell pellets (Figure S2). FUT8 protein levels were significantly higher in Gleason grade 7 and Gleason grade 8–10 (8+) tumours compared to Gleason grade 6 tumours (*p* < 0.01 and *p* < 0.0001) (Figure 1E) and in patients with metastasis compared to patients with localised disease (*p* = 0.0084) (Figure 1F). Taken together, our data suggest FUT8 is upregulated at both the gene and protein level in high grade prostate tumours and in patients with metastatic disease.

### 3.2 FUT8 Protein Levels Are Elevated in the Blood of Patients With Prostate Cancer

Shedding of glycosyltransferases from cells has previously been reported [86, 87, 88, 89] and we recently identified upregulation of the glycosyltransferase enzymes GALNT7 and ST6GAL1 in the blood of prostate cancer patients [58, 61]. We thus hypothesised that the FUT8 enzyme might also be detectable in serum/plasma samples from men with prostate cancer. Using pre‐validated sandwich ELISA assays (Figure S3), we monitored FUT8 protein levels in blood samples from men with prostate cancer. First, we tested FUT8 levels in plasma samples from 27 men with suspected prostate cancer. FUT8 levels were 3‐fold higher in plasma samples taken from men later diagnosed with prostate cancer compared to men given a ‘no cancer’ diagnosis (*n* = 27, *p* = 0.1364) (Figure 2A). Next, we monitored FUT8 plasma levels in 319 men diagnosed with either benign disease or prostate cancer. FUT8 protein levels were 2.1‐fold higher in men with prostate cancer compared to men with benign disease (*n* = 319, *p* < 0.05) (Figure 2B). We also detected higher levels of FUT8 protein in serum samples from men with high grade prostate cancer (Gleason grade 8–9) compared to low grade disease (Gleason grade 6–7) (*n* = 200, *p* < 0.0218) (Figure 2C). Finally, consistent with FUT8 being repressed by androgens, we detected significantly higher levels of FUT8 in matched serum samples taken from patients after ADT (*n* = 14, *p* = 0.002) (Figure 2D). Taken together, our findings show in addition to being upregulated in high grade prostate tumour tissue, the levels of FUT8 are also significantly higher in the blood of patients with aggressive disease.

### 3.3 FUT8 Promotes Prostate Tumour Growth, Cell Motility and Invasion

The data presented above show a link between FUT8 and aggressive prostate cancer.

**FIGURE 3:**
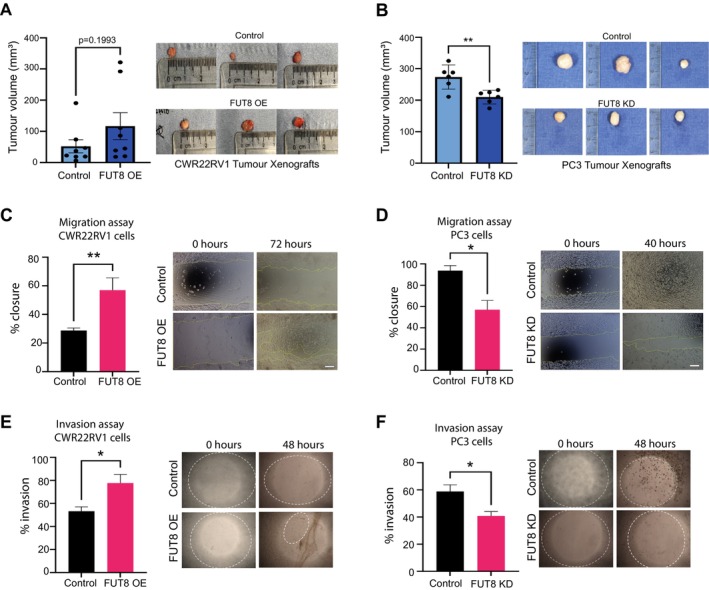
Upregulation of FUT8 in prostate cancer cells promotes tumour growth, migration and invasion. (A) Upregulation of FUT8 in CWR22Rv1 cells increases the growth of subcutaneous xenograft tumours. 1 × 10^7^ cells were injected into the flank of CD‐1 nude mice. Tumour size was measured every 3–4 days using callipers. Over 15 days the CWR22V1 tumours with overexpression of FUT8 were 2.23 folf bigger (*n* = 16, unpaired t test, *p* = 0.1993). Representative tumour images from each group are shown. (B) Knockdown of FUT8 using shRNA significantly reduces the growth of PC3 tumours in a subcutaneous xenograft model. 3 × 10^6^ PC3 cells were injected into the flank of NMRI mice. Tumour size was measured every 3–4 days using callipers. Over 40 days, the growth of PC3 tumours with knockdown of FUT8 was significantly reduced (*n* = 12, unpaired t test, *p* = 0.0055, **). (C‐F) Upregulation of FUT8 in CWR22Rv1 cells promotes cell migration (unpaired t text, *p* = 0.0092, **) and invasion (unpaired t test, *p* = 0.0156, *). Knockdown of FUT8 in PC3 cells decreases prostate cancer cell migration (unpaired t test, *p* = 0.0102, *) and invasion (unpaired t test, *p* = 0.0113, *). Scale bar is 20 μm.

Previous studies have linked FUT8 to the in vitro growth and motility of prostate cancer cells [84, 85, 90]. However, the impacts of FUT8 on prostate cancer biology have not yet been investigated in vivo. To address this, we created prostate cancer cells with stable overexpression (upregulation) or knockdown (downregulation) of FUT8 (Figure S3) and used these to study the effects of FUT8 on prostate cancer cell behaviour. Our findings show overexpression of FUT8 in CWR22Rv1 cells promotes proliferation and colony formation in vitro, whereas knockdown of FUT8 in PC3 and DU145 cells has the opposite effect (Figure S4). Next, using sub‐cutaneous xenograft models, we found that overexpression of FUT8 increased the growth of CWR22Rv1 tumours by 2.23 fold (*p* = 0.1993) (Figure 3A) whereas knockdown of FUT8 significantly suppressed the growth of PC3 tumours (*p* = 0.0055) (Figure 3B). Furthermore, in vitro assays showed that FUT8 can promote prostate cancer cell migration and invasion (Figure 3C–F). Taken together, the above data suggest that upregulation of FUT8 is linked to a more aggressive prostate cancer cell phenotype.

### 3.4 FUT8 Regulates Core Fucosylation of *N*‐Glycans in Prostate Cancer Cells

**FIGURE 4:**
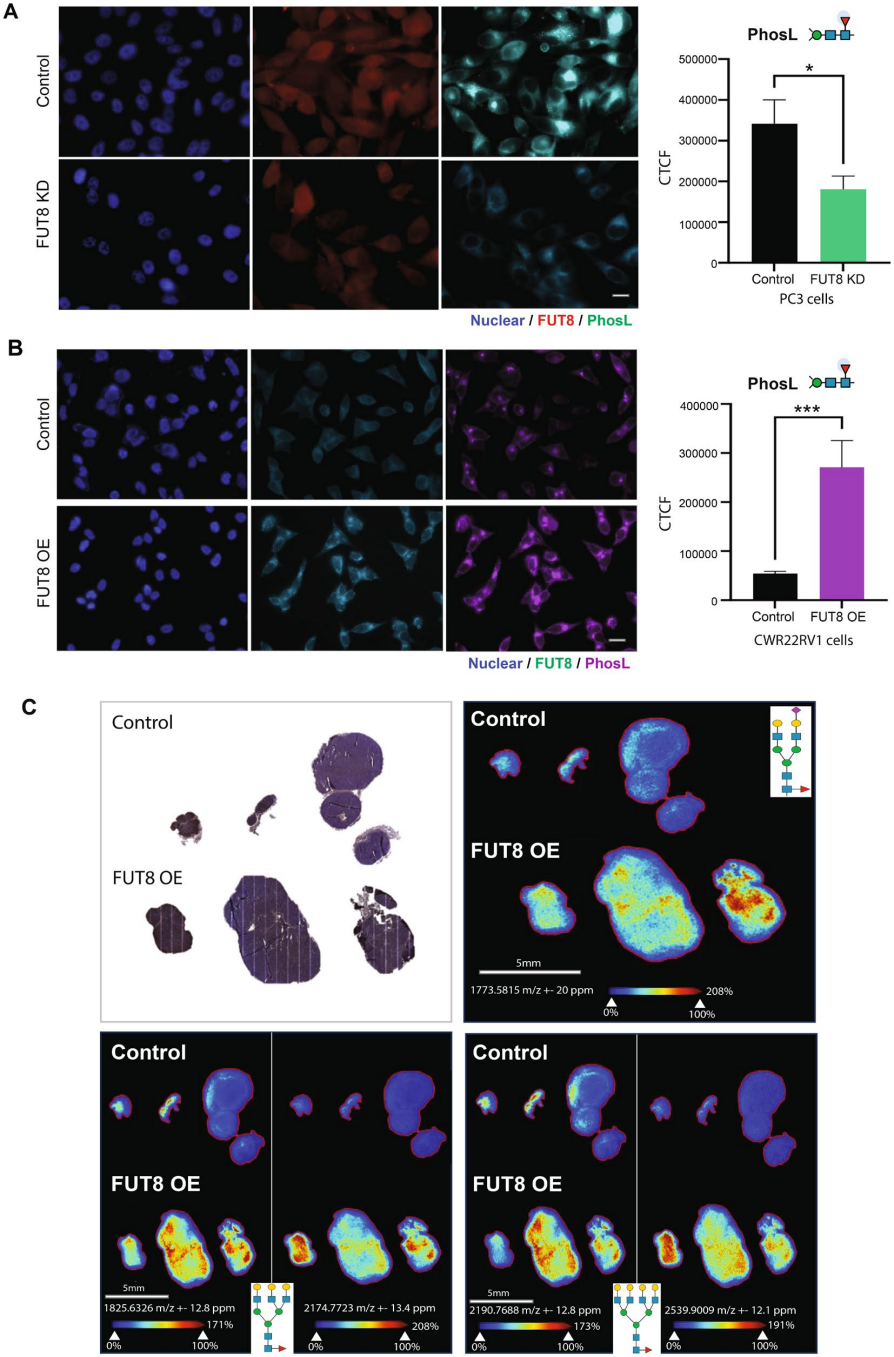
FUT8 mediates core fucosylation of *N*‐glycans in prostate cancer cells. (A,B) Detection of core fucosylated *N‐*glycans using PhoSL immunofluorescence. (A) PC3 cells with knockdown of FUT8 and have reduced levels of core fucosylated *N*‐glycans (unpaired t test, *p* = 0.0227, *) (B) CWR22Rv1 cells with overexpression of FUT8 have increased levels of core fucosylated *N*‐glycans (unpaired t test, *p* = 0.0005, ***). Scale bar = 10 μM. Corrected total cell fluorescence (CTCF) indicates a significant decrease in PhoSL binding intensity with FUT8 knockdown, while overexpression of FUT8 significantly increases PhoSL binding intensity. (C) Analysis of FUT8 protein and core‐fucosylated *N*‐glycans in CWR22Rv1 xenograft tumours (from the experiment shown in Figure 2A) using immunohistochemistry and *N‐*glycan Matrix‐assisted laser desorption/ionizationmass spectrometry imaging (MALDI‐MSI) to identify core‐fucosylated *N*‐glycans. Images show the spatial distribution of core fucosylated bi‐antennary *N*‐glycan (1773.581 m/z), tri‐antennary *N*‐glycan (1825.5961 m/z) and the complex core fucosylated tetra‐antennary *N‐*glycan (2190.7632 m/z). EndoF3 cleavage induced a shift of 349.137 amu. Glycan nomenclature: Blue square indicates GlcNAc, yellow circle indicates galactose, green circle indicates mannose, red triangle indicates fucose, and purple diamond indicates sialic acid. Scale bar is 5 mm.

The above data links FUT8 to high grade prostate cancer and a more aggressive tumour phenotype. To test if altered FUT8 expression changes the cell surface core fucosylation of prostate cancer cells, we utilised cell lines with knockdown or overexpression of FUT8.

(Figure S3). We then monitored recognition by the core fucose specific lectin Pholiota squarrosa (PhoSL), which binds exclusively to core α‐1,6‐fucosylated *N*‐glycans (and not other types of fucosylated oligosaccharides) [60, 91, 92]. Patterns of PhoSL immunofluorescence revealed that knockdown of FUT8 correlated with reduced levels of core fucosylation (Figure 4A and Figure S5A), whereas upregulation of FUT8 correlated with increased levels of core fucose (Figure 4B). This finding was confirmed via *N*‐glycan Matrix‐assisted laser desorption/ionisation mass spectrometry imaging (MALDI‐MSI) [71, 93]. Using an enzyme that specifically cleaves core‐fucosylated *N*‐glycans, endoglycosidase F3 [73], we show that increased levels of FUT8 in prostate tumours correlates with a core fucosylated *N*‐glycan structural theme (Figure 4C). Using PhoSL immunofluorescence, we also detected an abundance of core fucosylated *N*‐glycans in clinical prostate cancer tissue (Figure S5B). Together, this data indicates that upregulation of the fucosyltransferase FUT8 underpins the biosynthesis of malignant core fucosylated *N*‐glycans in prostate cancer cells.

### 3.5 Upregulation of FUT8 Alters Oncogenic Genes and Proteins in Prostate Cancer Cells

**FIGURE 5:**
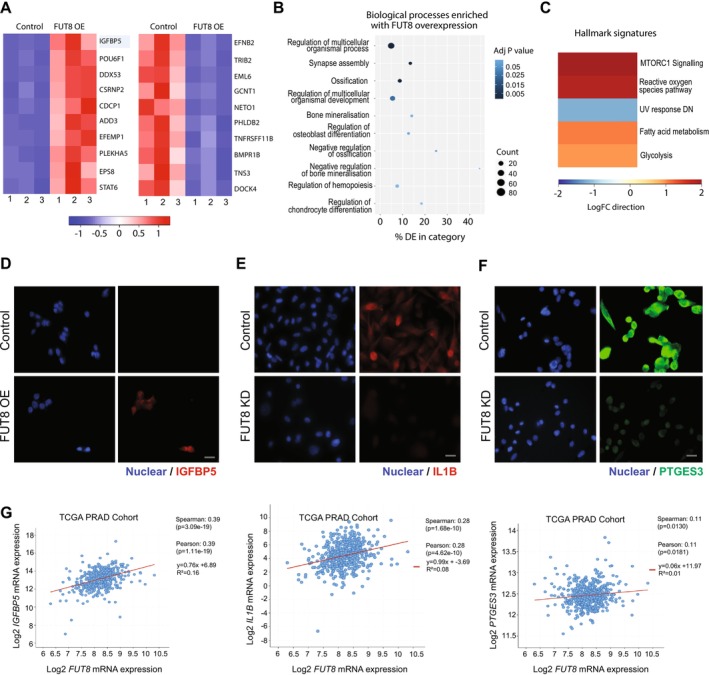
FUT8 regulates oncogenic genes and proteins in prostate cancer cells. RNA‐sequencing analysis of CWR22Rv1 cells with overexpression of FUT8 identified 381 differentially expressed genes (adjusted *p*‐value < 0.05, Log2FC 0.58) (Table S2). (A) Heatmap to illustrate the top 10 upregulated and 10 ten downregulated differentially expressed genes. (B, C) Gene Ontology and gene set enrichment analyses of genes regulated by FUT8 revealed CWR22Rv1 cells overexpressing FUT8 have enrichment in ‘ossification’, ‘bone mineralisation’ and ‘regulation of osteoblast differentiation’. (D–F) Validation at the protein level using immunocytochemistry shows (D) IGFBP5 is upregulated when FUT8 is overexpressed in CWR22Rv1 cells and (E, F) Knockdown of FUT8 downregulates IL1B and PTGES3 in PC3 cells. Scale bar is 20 μm. (G) Analysis of the TCGA PRAD cohort shows a significant correlation between the *FUT8* gene and levels of *IGFBP5*, *IL1B* and *PTGES3* in clinical prostate cancer tissue.

The findings presented above suggested overexpression of FUT8 is linked to high grade prostate cancer and can promote an aggressive cell phenotype. Next, to search for signalling networks regulated by FUT8 target glycoproteins, we used RNA‐sequencing to identify genes that change with either knockdown or overexpression of FUT8. Bioinformatic analysis identified 381 significant differentially expressed genes when FUT8 is overexpressed in CWR22Rv1 cells and 3519 significant differentially expressed genes when. FUT8 was depleted in PC3 cells (adjusted *p*‐value < 0.05, Log2FC 0.58) (Figure 5A, Figure S6 and Tables S2 and S3). Interestingly, Gene Ontology analysis revealed CWR22Rv1 cells overexpressing FUT8 have enrichment in ‘ossification’, ‘bone mineralisation’ and ‘regulation of osteoblast differentiation’, whereas PC3 cells with knockdown of FUT8 have enrichment in pathways related to the ‘immune system’, ‘cell migration’ and ‘adhesion’ (Figure 5B,C, Figure S6 and Tables S4 and S5). Validation at the protein level in prostate cancer cells confirmed a correlation between FUT8 and levels of insulin‐like growth factor binding protein‐5 (IGFBP5) (which is linked to prostate cancer progression [94]), interleukin 1 beta (IL1B) (a cytokine linked to an immune suppressive microenvironment [95]) and Prostaglandin E synthase 3 (PTGES3) (an AR regulator that promotes cell proliferation [96]) (Figure 5D–F). Furthermore, analysis of the TCGA PRAD cohort [62] revealed a significant correlation between expression of *FUT8* and genes for *IGFBP5*, *IL1B* and *PTGES3* in clinical prostate cancer tissue (Figure 5G). These findings provide novel insights into molecular mechanisms important for prostate cancer progression and point towards targeting FUT8 and/or its associated glycoproteins as novel targets for prostate cancer therapeutics.

### 3.6 Targeting FUT8 Activity With Fucosyltransferase Inhibitors Suppresses Prostate Tumour Growth

**FIGURE 6:**
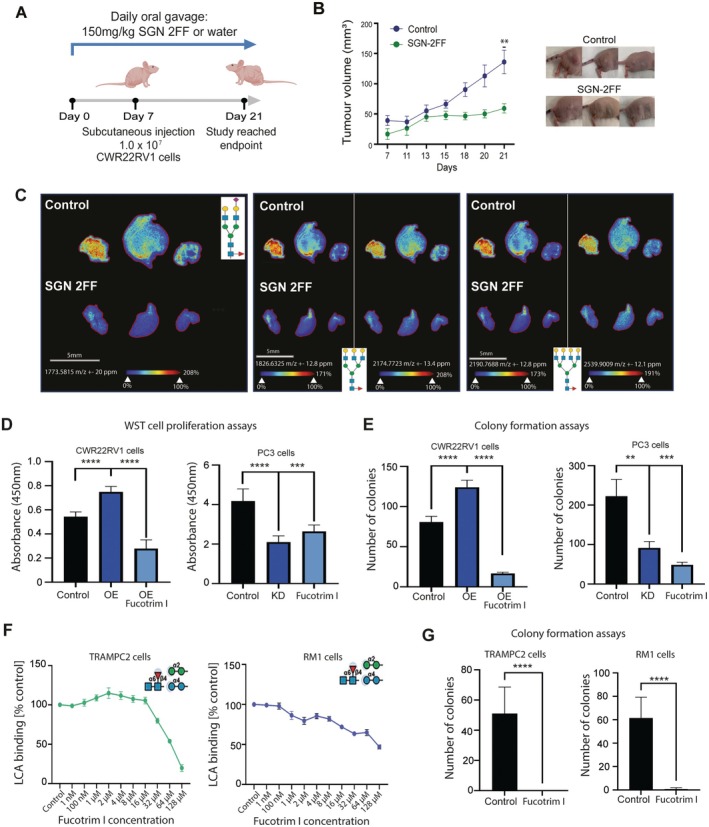
Targeting FUT8‐mediated core fucosylation in prostate cancer with fucosylation inhibitors suppresses tumour growth. (A) CWR22Rv1 cells were subcutaneously injected into the flank of 7‐week‐old CD‐1 nude mice. 7 days prior to implantations mice were randomised to start treatment with either 150 mg/kg fucosylation inhibitor SGN‐2FF or water via oral gavage daily (*n* = 10 mice/group). Tumour size was measured every 3–4 days using callipers. (B) Tumour volume (mm^3^) was significantly reduced in the SGN‐2FF treated mice after 21 days (Welch's *t*‐test for tumour volume on Day 21, *p* = 0.0034, **). Representative images of tumours are shown. (C) Analysis of CWR22Rv1 xenograft tumours (from experiment shown in Figure 6B) using *N*‐glycan MALDI‐MSI to identify core‐fucosylated *N*‐glycans. Images show the spatial distribution of core fucosylated bi‐antennary *N*‐glycan (1773.581 m/z), tri‐antennary *N*‐glycan (1825.5961 m/z) and the complex core fucosylated tetra‐antennary *N‐*glycan (2190.7632 m/z). EndoF3 cleavage induced a shift of 349.137 amu. Glycan nomenclature: blue square indicates GlcNAc, yellow circle indicates galactose, green circle indicates mannose, red triangle indicates fucose, and purple diamond indicates sialic acid. Scale bar is 5 mm. (D) WST‐1 cell proliferation assays show FUT8 overexpression significantly increases the proliferation of CWR22RV1 cells (unpaired *t*‐test, *p* < 0.0001, ****), and this is suppressed by treatment with 30 μM of Fucotrim I over 72 h (unpaired *t*‐test, *p* < 0.0001, ****). WST‐1 cell proliferation assays also show FUT8 knockdown significantly reduces the proliferation of PC3 cells (unpaired *t*‐test, *p* < 0.0001, ****) and by treatment with 30 μM of Fucotrim I for 72 h (unpaired *t*‐test, *p* = 0.0012, ***). (E) Colony formation assays show FUT8 overexpression significantly increases the ability of CWR22RV1 cells to survive and grow in colonies over 14 days (unpaired *t*‐test, *p* < 0.0001, ****), and this is suppressed by treatment with 30 μM Fucotrim I (unpaired *t* test, *p* < 0.0001, ****). PC3 cells with knockdown of FUT8 have reduced colony formation over 14 days (unpaired *t*‐test, *p* = 0.019, **). PC3 cells treated with 30 μM Fucotrim I for 14 days have reduced ability to survive and grow in colonies over 14 days (unpaired *t*‐test, *p* = 0.0015, ***). (F) Inhibition of fucosylation in TRAMPC2 and RM1 mouse prostate cancer cells Fucotrim I detected using LCA lectin flow cytometry (which recognises core fucosylated N‐glycans [103]. Cells were treated with a range of concentrations of Fucotrim I from 1 nM to 128 μM for 72 h. The mean fluorescence intensities were normalized to a DMSO control. (G) Colony formation assays show treatment with 64 μM Fucotrim significantly reduced cell colony formation for both TRAMPC2 cells (unpaired *t*‐test, *p* < 0.0001, ****) and RM1 cells (unpaired *t*‐test, *p* < 0.0001, ****) over 7 days.

To assess whether inhibition of FUT8 in prostate cancer cells will be clinically useful, we next chose to investigate whether systemic treatment with SGN‐2FF (a cell permeable fucosyltransferase inhibitor which has shown promising effects on tumour cells, immune cells, and the tumour microenvironment [97, 98, 99, 100, 101, 102]) can inhibit the in vivo growth of prostate tumours in mice. Daily oral gavage treatment with SGN‐2FF significantly suppressed the growth of CWR22Rv1 xenografts over 21 days (Figure 6A,B) and this was consistent with inhibition of core fucosylated *N*‐glycans in tumours (detected via MALDI‐MSI, where following EndoF3 treatment there was a shift of 349 m.u. for detected core fucosylated species) (Figure 6C). Although a Phase I clinical trial with SGN‐2FF for advanced solid tumours produced a significant drop in tumour burden, the study was terminated due to safety concerns (NCT 02952989) [104]. However, the efficacy of SGN‐2FF and its promise as a cancer therapeutic has inspired the development of new fucosylation inhibitors with higher potency than SGN‐2FF, including the SGN‐2FF derivatives A2FF1P and B2FF1P, and the metabolic inhibitors Fucotrim I and II [91, 105, 106]. Previously, we showed these compounds can effectively shut down the synthesis of fucosylated glycans in prostate cancer cells to remodel the prostate cancer glycome with only minor apparent side effects on other glycan types [74]. Using concentrations previously optimised by us for use on prostate cancer cells [74], we next tested if potent metabolic fucosylation inhibitors can inhibit the activity of FUT8 in prostate cancer. We show that treatment with A2FF1P, B2FF1P, or Fucotrim I/II significantly reduces the proliferation and survival of CWR22Rv1 cells with upregulation of FUT8 (Figure 6D,E and Figure S7). As our previous study identified Fucotrim I as having the highest efficacy for prostate cancer cells [74], we next chose to test this inhibitor using additional prostate cancer models. Treatment with Fucotrim I suppressed the growth of PC3 cells to a similar level as FUT8 knockdown (Figure 6D,E). Furthermore, our findings show Fucotrim I can block fucose incorporation and suppress colony formation in mouse prostate cancer cell lines (Figure 6F,G and Figure S8). Taken together, our data shows that blocking fucosylation inhibits the growth of prostate tumours and highlights the potential therapeutic use of fucosylation inhibitors (once modifications render them more targeted towards cancer cells) to block the malignant action of FUT8 in prostate cancer.

## 4 Discussion

Altered core fucosylation mediated by FUT8 is a key change in tumour glycan patterns that contributes to cancer growth, metastasis, and immune evasion [22, 25, 91, 107]. In this study, we measured the levels of FUT8 in > 1500 clinical samples across multiple patient cohorts and verify upregulation of FUT8 in high grade tumours and in patients with metastasis, and further show that the levels of blood borne FUT8 are also increased in patients with aggressive disease. Our findings show FUT8 underpins the synthesis of malignant core fucosylated *N*‐glycans in prostate cancer cells and functionally links FUT8 with prostate tumour growth and the regulation of genes and pathways implicated in disease progression. Furthermore, we find that blocking the activity of FUT8 using fucosylation inhibitors can suppress the growth of prostate tumours. Based on these findings, we propose FUT8‐mediated core fucosylation regulates pro‐oncogenic mechanisms involved in prostate cancer progression and this can likely be exploited for therapeutic usage.

Targeting aberrant fucosylation holds huge potential for cancer research, and given the critical roles of FUT8 in tumour pathology, it is poised to be a druggable target for new cancer therapies [107]. The fucosylation inhibitor SGN‐2FF has demonstrated promising anti‐cancer effects on tumour cells, immune cells and the tumour microenvironment [22, 74, 97, 98, 99, 100, 102, 108]. However, although a Phase I clinical trial with SGN‐2FF produced promising findings for advanced solid tumours, the study was terminated early due to safety issues (NCT 02952989) [104]. Since then, a range of additional fucosylation inhibitors have been developed [109], and have begun to show promise for treating cancer [74]. Here, we find that blocking fucosylation using SGN‐2FF can suppress the in vivo growth of prostate tumours. Furthermore, using in vitro assays, we find treatment with next generation fucosylation inhibitors (which reach higher effective concentrations within the cell [98, 99]) can inhibit the activity of FUT8 in prostate cancer cells. Our study provides proof‐of principle data to show metabolic inhibitors of fucosylation can be used to target FUT8‐mediated core fucosylation to reduce prostate cancer cell growth and highlights the potential to utilise these type of inhibitors as new therapies for prostate cancer.

As all fucosyltransferases use GDP‐fucose as a substrate, inhibiting global fucosylation will block all fucose containing glycans (and not just those containing core fucose) which could lead to unwanted off target side effects. Recent advances in deciphering the crystal structure of FUT8 [29, 107] have led to the development of selective FUT8 inhibitors using rationally optimised compounds in combination with virtual screening techniques. FDW028 is highly selective small‐molecule inhibitor of FUT8 identified through virtual screening and chemical refinement to bind the GDP‐fucose pocket that exhibits potent anti‐tumour activity by defucosylation, has demonstrated in vivo efficacy when applied locally near tumours, and can prolong the survival of mice with metastatic colorectal cancer [110, 111]. Manabe et al. have reported a GDP‐dependent covalent inhibitor that functions in cells without mimicking the donor substrate [112], and Gilormini et al. have introduced β‐carbafucose, a non‐selective metabolic inhibitor that targets several fucosyltransferases, including FUT8. Notably, β‐carbafucose treatment led to a marked increase in antibody‐dependent cellular cytotoxicity (ADCC) by producing afucosylated IgG in vivo, which is highly desirable for therapeutic antibody enhancement [113]. To date, no compensatory mechanisms have been described that restore core α1,6‐fucosylation in the absence of FUT8. This enzyme appears to be functionally unique, and its loss leads to profound phenotypic effects, including disrupted receptor signalling and developmental defects, without evidence of redundancy by other fucosyltransferases [114, 115]. In published in vivo studies, no significant toxic effects have been observed with the use of FDW028. Specifically, treated mice exhibited no notable changes in body weight, and no cytotoxicity was detected in cell‐based assays, suggesting a favourable tolerability profile at therapeutic doses [110]. Further studies will of course be needed to assess long‐term safety, but current data are encouraging. Moving forward, we anticipate these specific FUT8 inhibitors will be relevant for prostate cancer therapy and are candidates for further investigation.

Core fucosylation is reported to occur in 20–90% of proteins, including cytokines, receptors and immune checkpoint molecules, influencing their cellular function and playing an important role in the tumour immune microenvironment (TIME) [25, 91]. Studies have identified a role for FUT8 mediated core fucosylation in regulating epidermal growth factor receptor (EGFR) [85, 116], transforming growth factor beta receptor 1 (TGFBR1) [33], E‐cadherin [39, 117], and the immune checkpoint molecules programmed cell death receptor 1 (PD‐1) and B7‐H3 [46, 118]. A recent study utilised MALDI‐IMS to document *N*‐glycome alterations in tumours and revealed that while total core fucosylation levels do not change in prostate cancer relative to normal prostate tissue, it is likely the specific *N*‐glycans being core fucosylated are the important factor [72]. Here, we show upregulation of FUT8 correlates with the expression of oncogenic proteins, including IGFBP5, IL1B and PTGES3, which have been functionally linked to disease progression [94, 95, 96]. Our findings are consistent with a previous study which revealed FUT8 is a master regulator of cell surface receptors in aggressive prostate tumours and can promote cell survival in androgen depleted conditions [85]. While the full repertoire of specific *N*‐glycoproteins modified by FUT8 in prostate cancer are likely still to be fully discovered, it is clear the role of FUT8 is multi‐faceted (and likely involves the regulation of cell signalling receptors, cytokines and immune checkpoint molecules). Aberrant core fucosylation of tumours has been functionally implicated in tumour immune evasion and metastasis [22, 25, 41] but this has not yet been investigated for prostate cancer. Our data identifies correlations between FUT8 levels and pathways including ‘regulation of osteoblast differentiation’ and ‘immune response’. Metastasis to bone is common in prostate cancer and osteoblasts (bone‐forming cells) are implicated in this process [119]. FUT8 maintains high expression and protein stability of immune checkpoint molecules (including PD1, PDL1, PDL1 and B7H3) meaning there is a functional link between FUT8 activity and the suppressive state of the tumour microenvironment [46, 118, 120], and FUT8 is now a central target for cancer immunotherapy [91]. Future studies could investigate how upregulation of FUT8 in aggressive prostate cancer impacts both bone metastasis and the tumour immune microenvironment (including regulation of osteoblasts and immune checkpoint molecules). Further deciphering the biological effects mediated by FUT8 in prostate cancer could lead to new strategies targeting the FUT8 immune checkpoint axis to improving anti‐tumour immune responses in patients with cancer.

In summary, we report FUT8 is upregulated in high grade prostate tumours, and this is linked to a more aggressive tumour phenotype. Mechanistically, we show FUT8 regulates malignant core fucosylated *N*‐glycans on prostate cancer cells and is correlated with the expression of oncogenic proteins and pathways linked to disease progression. Furthermore, we find FUT8‐mediated core fucosylation can be targeted using metabolic fucosylation inhibitors, and that this suppresses the growth of prostate tumours. Our study cements FUT8 as an important driver of prostate cancer progression and points to the need for further characterisation of core fucosylation in prostate tumours. Given the critical roles of FUT8 in prostate cancer biology, it is poised to be a druggable target for cancer therapy. Moving forward, we propose that both global fucosylation and small molecule inhibitors of FUT8 are relevant to patients with prostate cancer and should be explored as new therapeutic avenues.

## Author Contributions

K.B., M.O.‐M., K.H., E.A.V., H.S., O.H. and Z.P. performed in vitro experiments. K.B., E.S. and H.T. performed the in vivo studies. F.F., B.K., P.M., J.M., M.C., L.H. and N.J.M. contributed to clinical sample collection. K.B. and L.W. performed IHC on tissue sections. K.B. and L.W. scored pathology sections. K.B., G.G. and R.R.D. performed N‐glycan MALDI‐IMS. K.H., M.O.‐M. and K.B. carried out bioinformatics analyses. E.D.G.‐B. provided PhoSL lectin for use in the study and assisted with study design. E.R., J.F.A.P. and N.E. provided Fucotrim I for use in the study and assisted with study design and data analysis. K.B., J.M., M.O.‐M., R.R.D., R.H.G. and E.S. designed, analysed and interpreted the study. J.M. wrote the manuscript and created the figures. R.R.D., D.J.E., R.H., R.H.G., T.J.B., R.R.D. and N.W. contributed to critical review and paper writing. J.M. conceived the study and is senior author and corresponding author. All authors read the manuscript, agreed with the content, and were given the opportunity to provide input.

## Conflicts of Interest

J.M. and E.S. are shareholders of GlycoScoreDx Ltd. and have filed patents related to this work (GB Patent GB2,594,103 and US Patent App. 17/780,508). J.F.A.P. and E.R. are shareholders of and employed by GlycoTherapeutics B.V. T.J.B. is a shareholder of and scientific advisor of GlycoTherapeutics B.V.; J.F.A.P. and T.J.B. are shareholders of Synvenio B.V. Radboud University and Radboudumc have filed patent applications related to Fucotrim I and Fucotrim II. All other authors declare no conflicts of interest.

## Supporting information


Figure S1.
Figure S2.
Figure S3.
Figure S4.
Figure S5.
Figure S6.
Figure S7.
Figure S8.

Data S1.

## Data Availability

The data that supports the findings of this study are available in the [Link], [Link] of this article.

## References

[1] H. Sung , J. Ferlay , R. L. Siegel , et al., “Global Cancer Statistics 2020: GLOBOCAN Estimates of Incidence and Mortality Worldwide for 36 Cancers in 185 Countries,” CA: A Cancer Journal for Clinicians 71, no. 3 (2021): 209–249. 33538338 10.3322/caac.21660

[2] F. Bray , M. Laversanne , H. Sung , et al., “Global Cancer Statistics 2022: GLOBOCAN Estimates of Incidence and Mortality Worldwide for 36 Cancers in 185 Countries,” CA: A Cancer Journal for Clinicians 74, no. 3 (2024): 229–263. 38572751 10.3322/caac.21834

[3] E. Archer Goode , N. Wang , and J. Munkley , “Prostate Cancer Bone Metastases Biology and Clinical Management (Review),” Oncology Letters 25, no. 4 (2023): 163. 36960185 10.3892/ol.2023.13749 PMC10028493

[4] P. Nuhn , J. S. De Bono , K. Fizazi , et al., “Update on Systemic Prostate Cancer Therapies: Management of Metastatic Castration‐Resistant Prostate Cancer in the Era of Precision Oncology,” European Urology 75, no. 1 (2019): 88–99. 29673712 10.1016/j.eururo.2018.03.028

[5] J. Mateo , K. Fizazi , S. Gillessen , et al., “Managing Nonmetastatic Castration‐Resistant Prostate Cancer,” European Urology 75, no. 2 (2019): 285–293. 30119985 10.1016/j.eururo.2018.07.035

[6] E. L. Karen , M. Jennifer , and J. E. David , “Androgen receptor and prostate cancer,” AIMS Molecular Science 3, no. 2 (2016): 280–299.

[7] D. N. Rodrigues , G. Boysen , S. Sumanasuriya , G. Seed , A. M. Marzo , and J. de Bono , “The Molecular Underpinnings of Prostate Cancer: Impacts on Management and Pathology Practice,” Journal of Pathology 241, no. 2 (2017): 173–182. 27753448 10.1002/path.4826

[8] Y. Zong and A. S. Goldstein , “Adaptation or Selection—Mechanisms of Castration‐Resistant Prostate Cancer,” Nature Reviews Urology 10, no. 2 (2013): 90–98. 23247694 10.1038/nrurol.2012.237

[9] M. A. Rice , S. V. Malhotra , and T. Stoyanova , “Second‐Generation Antiandrogens: From Discovery to Standard of Care in Castration Resistant Prostate Cancer,” Frontiers in Oncology 9 (2019): 801. 31555580 10.3389/fonc.2019.00801 PMC6723105

[10] J. E. Vellky and W. A. Ricke , “Development and Prevalence of Castration‐Resistant Prostate Cancer Subtypes,” Neoplasia 22, no. 11 (2020): 566–575. 32980775 10.1016/j.neo.2020.09.002 PMC7522286

[11] J. J. Adashek , R. K. Jain , and J. Zhang , “Clinical Development of PARP Inhibitors in Treating Metastatic Castration‐Resistant Prostate Cancer,” Cells 8, no. 8 (2019): 860. 31404966 10.3390/cells8080860 PMC6721701

[12] T. M. Amaral , D. Macedo , I. Fernandes , and L. Costa , “Castration‐Resistant Prostate Cancer: Mechanisms, Targets, and Treatment,” Prostate Cancer 2012 (2012): 327253. 22530130 10.1155/2012/327253 PMC3316959

[13] L. Dong , R. C. Zieren , W. Xue , T. M. de Reijke , and K. J. Pienta , “Metastatic Prostate Cancer Remains Incurable, Why?,” Asian Journal of Urology 6, no. 1 (2019): 26–41. 30775246 10.1016/j.ajur.2018.11.005 PMC6363601

[14] M. Cerasuolo , F. Maccarinelli , D. Coltrini , et al., “Modeling Acquired Resistance to the Second‐Generation Androgen Receptor Antagonist Enzalutamide in the TRAMP Model of Prostate Cancer,” Cancer Research 80, no. 7 (2020): 1564–1577. 32029552 10.1158/0008-5472.CAN-18-3637

[15] J. Munkley and D. J. Elliott , “Hallmarks of Glycosylation in Cancer,” Oncotarget 7, no. 23 (2016): 35478–35489. 27007155 10.18632/oncotarget.8155 PMC5085245

[16] B. N. Vajaria and P. S. Patel , “Glycosylation: A Hallmark of Cancer?,” Glycoconjugate Journal 34, no. 2 (2017): 147–156. 27975160 10.1007/s10719-016-9755-2

[17] S. S. Pinho and C. A. Reis , “Glycosylation in Cancer: Mechanisms and Clinical Implications,” Nature Reviews. Cancer 15, no. 9 (2015): 540–555. 26289314 10.1038/nrc3982

[18] B. A. H. Smith and C. R. Bertozzi , “The Clinical Impact of Glycobiology: Targeting Selectins, Siglecs and Mammalian Glycans,” Nature Reviews. Drug Discovery 20, no. 3 (2021): 217–243. 33462432 10.1038/s41573-020-00093-1 PMC7812346

[19] S. Mereiter , M. Balmana , D. Campos , J. Gomes , and C. A. Reis , “Glycosylation in the Era of Cancer‐Targeted Therapy: Where Are we Heading?,” Cancer Cell 36, no. 1 (2019): 6–16. 31287993 10.1016/j.ccell.2019.06.006

[20] A. F. Costa , D. Campos , C. A. Reis , and C. Gomes , “Targeting Glycosylation: A New Road for Cancer Drug Discovery,” Trends Cancer 6, no. 9 (2020): 757–766. 32381431 10.1016/j.trecan.2020.04.002

[21] D. J. Becker and J. B. Lowe , “Fucose: Biosynthesis and Biological Function in Mammals,” Glycobiology 13, no. 7 (2003): 41R–53R. 10.1093/glycob/cwg054 12651883

[22] K. Bastian , E. Scott , D. J. Elliott , and J. Munkley , “FUT8 Alpha‐(1,6)‐Fucosyltransferase in Cancer,” International Journal of Molecular Sciences 22, no. 1 (2021): 455. 33466384 10.3390/ijms22010455 PMC7795606

[23] J. Li , H. C. Hsu , J. D. Mountz , and J. G. Allen , “Unmasking Fucosylation: From Cell Adhesion to Immune System Regulation and Diseases,” Cell Chemical Biology 25, no. 5 (2018): 499–512. 29526711 10.1016/j.chembiol.2018.02.005

[24] E. Miyoshi , K. Moriwaki , and T. Nakagawa , “Biological Function of Fucosylation in Cancer Biology,” Journal of Biochemistry 143, no. 6 (2008): 725–729. 18218651 10.1093/jb/mvn011

[25] M. Shi , X. R. Nan , and B. Q. Liu , “The Multifaceted Role of FUT8 in Tumorigenesis: From Pathways to Potential Clinical Applications,” International Journal of Molecular Sciences 25, no. 2 (2024): 1068. 38256141 10.3390/ijms25021068 PMC10815953

[26] N. Uozumi , S. Yanagidani , E. Miyoshi , et al., “Purification and cDNA Cloning of Porcine Brain GDP‐L‐Fuc:N‐Acetyl‐Beta‐D‐Glucosaminide alpha1‐‐>6fucosyltransferase,” Journal of Biological Chemistry 271, no. 44 (1996): 27810–27817. 8910378 10.1074/jbc.271.44.27810

[27] E. Miyoshi , K. Noda , Y. Yamaguchi , et al., “The alpha1‐6‐Fucosyltransferase Gene and Its Biological Significance,” Biochimica et Biophysica Acta 1473, no. 1 (1999): 9–20. 10580126 10.1016/s0304-4165(99)00166-x

[28] M. Schneider , E. Al‐Shareffi , and R. S. Haltiwanger , “Biological Functions of Fucose in Mammals,” Glycobiology 27, no. 7 (2017): 601–618. 28430973 10.1093/glycob/cwx034 PMC5458543

[29] A. García‐García , L. Ceballos‐Laita , S. Serna , et al., “Structural Basis for Substrate Specificity and Catalysis of α1,6‐Fucosyltransferase,” Nature Communications 11, no. 1 (2020): 973. 10.1038/s41467-020-14794-z PMC7033129 32080177

[30] Q. Yang and L. X. Wang , “Mammalian Alpha‐1,6‐Fucosyltransferase (FUT8) is the Sole Enzyme Responsible for the N‐Acetylglucosaminyltransferase I‐Independent Core Fucosylation of High‐Mannose N‐Glycans,” Journal of Biological Chemistry 291, no. 21 (2016): 11064–11071. 27008861 10.1074/jbc.M116.720789 PMC4900256

[31] X. Wang , S. Inoue , J. Gu , et al., “Dysregulation of TGF‐beta1 Receptor Activation Leads to Abnormal Lung Development and Emphysema‐Like Phenotype in Core Fucose‐Deficient Mice,” Proceedings of the National Academy of Sciences of the United States of America 102, no. 44 (2005): 15791–15796. 16236725 10.1073/pnas.0507375102 PMC1257418

[32] N. Yamane‐Ohnuki , S. Kinoshita , M. Inoue‐Urakubo , et al., “Establishment of FUT8 Knockout Chinese Hamster Ovary Cells: An Ideal Host Cell Line for Producing Completely Defucosylated Antibodies With Enhanced Antibody‐Dependent Cellular Cytotoxicity,” Biotechnology and Bioengineering 87, no. 5 (2004): 614–622. 15352059 10.1002/bit.20151

[33] C. Y. Chen , Y. H. Jan , Y. H. Juan , et al., “Fucosyltransferase 8 as a Functional Regulator of Nonsmall Cell Lung Cancer,” Proceedings of the National Academy of Sciences of the United States of America 110, no. 2 (2013): 630–635. 23267084 10.1073/pnas.1220425110 PMC3545778

[34] R. Honma , I. Kinoshita , E. Miyoshi , et al., “Expression of Fucosyltransferase 8 Is Associated With an Unfavorable Clinical Outcome in Non‐Small Cell Lung Cancers,” Oncology 88, no. 5 (2015): 298–308. 25572677 10.1159/000369495

[35] F. Li , S. Zhao , Y. Cui , et al., “α1,6‐Fucosyltransferase (FUT8) Regulates the Cancer‐Promoting Capacity of Cancer‐Associated Fibroblasts (CAFs) by Modifying EGFR Core Fucosylation (CF) in Non‐Small Cell Lung Cancer (NSCLC),” American Journal of Cancer Research 10, no. 3 (2020): 816–837. 32266093 PMC7136908

[36] K. Noda , E. Miyoshi , N. Uozumi , et al., “Gene Expression of α1‐6 Fucosyltransferase in Human Hepatoma Tissues: A Possible Implication for Increased Fucosylation of α‐Fetoprotein,” Hepatology 28, no. 4 (1998): 944–952. 9755230 10.1002/hep.510280408

[37] L. Muinelo‐Romay , C. Vázquez‐Martín , S. Villar‐Portela , E. Cuevas , E. Gil‐Martín , and A. Fernández‐Briera , “Expression and Enzyme Activity of Alpha(1,6)fucosyltransferase in Human Colorectal Cancer,” International Journal of Cancer 123, no. 3 (2008): 641–646. 18491404 10.1002/ijc.23521

[38] M. Noda , H. Okayama , Y. Kofunato , et al., “Prognostic Role of FUT8 Expression in Relation to p53 Status in Stage II and III Colorectal Cancer,” PLoS One 13, no. 7 (2018): e0200315. 29975776 10.1371/journal.pone.0200315 PMC6033451

[39] D. Osumi , M. Takahashi , E. Miyoshi , et al., “Core Fucosylation of E‐Cadherin Enhances Cell‐Cell Adhesion in Human Colon Carcinoma WiDr Cells,” Cancer Science 100, no. 5 (2009): 888–895. 19302290 10.1111/j.1349-7006.2009.01125.x PMC11159289

[40] Y. Ito , A. Miyauchi , H. Yoshida , et al., “Expression of alpha1,6‐Fucosyltransferase (FUT8) in Papillary Carcinoma of the Thyroid: Its Linkage to Biological Aggressiveness and Anaplastic Transformation,” Cancer Letters 200, no. 2 (2003): 167–172. 14568171 10.1016/s0304-3835(03)00383-5

[41] P. Agrawal , B. Fontanals‐Cirera , E. Sokolova , et al., “A Systems Biology Approach Identifies FUT8 as a Driver of Melanoma Metastasis,” Cancer Cell 31, no. 6 (2017): 804–819. 28609658 10.1016/j.ccell.2017.05.007 PMC5649440

[42] K. Tada , M. Ohta , S. Hidano , et al., “Fucosyltransferase 8 Plays a Crucial Role in the Invasion and Metastasis of Pancreatic Ductal Adenocarcinoma,” Surgery Today 50, no. 7 (2020): 767–777. 31950256 10.1007/s00595-019-01953-z

[43] X. Lv , J. Song , K. Xue , et al., “Core Fucosylation of Copper Transporter 1 Plays a Crucial Role in Cisplatin‐Resistance of Epithelial Ovarian Cancer by Regulating Drug Uptake,” Molecular Carcinogenesis 58, no. 5 (2019): 794–807. 30614075 10.1002/mc.22971

[44] T. Takahashi , Y. Ikeda , E. Miyoshi , Y. Yaginuma , M. Ishikawa , and N. Taniguchi , “alpha1,6fucosyltransferase Is Highly and Specifically Expressed in Human Ovarian Serous Adenocarcinomas,” International Journal of Cancer 88, no. 6 (2000): 914–919. 11093814 10.1002/1097-0215(20001215)88:6<914::aid-ijc12>3.0.co;2-1

[45] C. F. Tu , M. Y. Wu , Y. C. Lin , R. Kannagi , and R. B. Yang , “FUT8 Promotes Breast Cancer Cell Invasiveness by Remodeling TGF‐β Receptor Core Fucosylation,” Breast Cancer Research 19, no. 1 (2017): 111. 28982386 10.1186/s13058-017-0904-8 PMC5629780

[46] Y. Huang , H. L. Zhang , Z. L. Li , et al., “FUT8‐Mediated Aberrant N‐Glycosylation of B7H3 Suppresses the Immune Response in Triple‐Negative Breast Cancer,” Nature Communications 12, no. 1 (2021): 2672. 10.1038/s41467-021-22618-x PMC8113546 33976130

[47] C. F. Tu , F. A. Li , L. H. Li , and R. B. Yang , “Quantitative Glycoproteomics Analysis Identifies Novel FUT8 Targets and Signaling Networks Critical for Breast Cancer Cell Invasiveness,” Breast Cancer Research 24, no. 1 (2022): 21. 35303925 10.1186/s13058-022-01513-3 PMC8932202

[48] L. Yue , C. Han , Z. Li , et al., “Fucosyltransferase 8 Expression in Breast Cancer Patients: A High Throughput Tissue Microarray Analysis,” Histology and Histopathology 31, no. 5 (2016): 547–555. 26596733 10.14670/HH-11-693

[49] X. Wang , J. Chen , Q. K. Li , et al., “Overexpression of α (1,6) Fucosyltransferase Associated With Aggressive Prostate Cancer,” Glycobiology 24, no. 10 (2014): 935–944. 24906821 10.1093/glycob/cwu051 PMC4153758

[50] N. Höti , S. Yang , Y. Hu , P. Shah , M. C. Haffner , and H. Zhang , “Overexpression of α (1,6) Fucosyltransferase in the Development of Castration‐Resistant Prostate Cancer Cells,” Prostate Cancer and Prostatic Diseases 21, no. 1 (2018): 137–146. 29339807 10.1038/s41391-017-0016-7 PMC5895601

[51] K. Fujita , K. Hatano , E. Tomiyama , et al., “Serum Core‐Type Fucosylated Prostate‐Specific Antigen Index for the Detection of High‐Risk Prostate Cancer,” International Journal of Cancer 148, no. 12 (2021): 3111–3118. 33594666 10.1002/ijc.33517

[52] K. Hatano , T. Yoneyama , S. Hatakeyama , et al., “Simultaneous Analysis of Serum α2,3‐Linked Sialylation and Core‐Type Fucosylation of Prostate‐Specific Antigen for the Detection of High‐Grade Prostate Cancer,” British Journal of Cancer 126, no. 5 (2022): 764–770. 34802050 10.1038/s41416-021-01637-x PMC8888746

[53] S. Gilgunn , P. J. Conroy , R. Saldova , P. M. Rudd , and R. J. O'Kennedy , “Aberrant PSA Glycosylation—A Sweet Predictor of Prostate Cancer,” Nature Reviews Urology 10, no. 2 (2013): 99–107. 23318363 10.1038/nrurol.2012.258

[54] E. Llop , M. Ferrer‐Batalle , S. Barrabes , et al., “Improvement of Prostate Cancer Diagnosis by Detecting PSA Glycosylation‐Specific Changes,” Theranostics 6, no. 8 (2016): 1190–1204. 27279911 10.7150/thno.15226 PMC4893645

[55] S. Halldórsson , L. Hillringhaus , C. Hojer , et al., “Development of a First‐In‐Class Antibody and a Specific Assay for α‐1,6‐Fucosylated Prostate‐Specific Antigen,” Scientific Reports 14, no. 1 (2024): 16512. 39020051 10.1038/s41598-024-67545-1 PMC11254934

[56] K. Fujita , M. Shimomura , M. Uemura , et al., “Serum Fucosylated Haptoglobin as a Novel Prognostic Biomarker Predicting High‐Gleason Prostate Cancer,” Prostate 74, no. 10 (2014): 1052–1058. 24802742 10.1002/pros.22824

[57] J. Munkley , L. Li , S. R. G. Krishnan , et al., “Androgen‐Regulated Transcription of ESRP2 Drives Alternative Splicing Patterns in Prostate Cancer,” eLife 8 (2019): 8. 10.7554/eLife.47678 PMC6788855 31478829

[58] E. Scott , K. Hodgson , B. Calle , et al., “Upregulation of GALNT7 in Prostate Cancer Modifies O‐Glycosylation and Promotes Tumour Growth,” Oncogene 42, no. 12 (2023): 926–937. 36725887 10.1038/s41388-023-02604-x PMC10020086

[59] E. A. Goode , M. Orozco‐Moreno , K. Hodgson , et al., “Sialylation Inhibition Can Partially Revert Acquired Resistance to Enzalutamide in Prostate Cancer Cells,” Cancers (Basel) 16, no. 17 (2024): 2953. 39272811 10.3390/cancers16172953 PMC11393965

[60] Y. Kobayashi , H. Tateno , H. Dohra , et al., “A Novel Core Fucose‐Specific Lectin From the Mushroom Pholiota Squarrosa,” Journal of Biological Chemistry 287, no. 41 (2012): 33973–33982. 22872641 10.1074/jbc.M111.327692 PMC3464508

[61] E. Scott , E. Archer Goode , R. Garnham , et al., “ST6GAL1‐Mediated Aberrant Sialylation Promotes Prostate Cancer Progression,” Journal of Pathology 261, no. 1 (2023): 71–84. 37550801 10.1002/path.6152

[62] Cancer Genome Atlas Research N , “The Molecular Taxonomy of Primary Prostate Cancer,” Cell 163, no. 4 (2015): 1011–1025. 26544944 10.1016/j.cell.2015.10.025 PMC4695400

[63] C. Gerhauser , F. Favero , T. Risch , et al., “Molecular Evolution of Early‐Onset Prostate Cancer Identifies Molecular Risk Markers and Clinical Trajectories,” Cancer Cell 34, no. 6 (2018): 996–1011. 30537516 10.1016/j.ccell.2018.10.016 PMC7444093

[64] E. Cerami , J. Gao , U. Dogrusoz , et al., “The cBio Cancer Genomics Portal: An Open Platform for Exploring Multidimensional Cancer Genomics Data,” Cancer Discovery 2, no. 5 (2012): 401–404. 22588877 10.1158/2159-8290.CD-12-0095 PMC3956037

[65] J. Gao , B. A. Aksoy , U. Dogrusoz , et al., “Integrative Analysis of Complex Cancer Genomics and Clinical Profiles Using the cBioPortal,” Science Signaling 6, no. 269 (2013): pl1. 23550210 10.1126/scisignal.2004088 PMC4160307

[66] J. Munkley , D. Vodak , K. E. Livermore , et al., “Glycosylation Is an Androgen‐Regulated Process Essential for Prostate Cancer Cell Viability,” eBioMedicine 8 (2016): 103–116. 27428423 10.1016/j.ebiom.2016.04.018 PMC4919605

[67] S. M. Walker , L. A. Knight , A. M. McCavigan , et al., “Molecular Subgroup of Primary Prostate Cancer Presenting With Metastatic Biology,” European Urology 72, no. 4 (2017): 509–518. 28408174 10.1016/j.eururo.2017.03.027

[68] M. Nouri , S. Massah , J. Caradec , et al., “Transient Sox9 Expression Facilitates Resistance to Androgen‐Targeted Therapy in Prostate Cancer,” Clinical Cancer Research 26, no. 7 (2020): 1678–1689. 31919137 10.1158/1078-0432.CCR-19-0098

[69] K. Hodgson , M. Orozco‐Moreno , E. A. Goode , et al., “Sialic Acid Blockade Inhibits the Metastatic Spread of Prostate Cancer to Bone,” eBioMedicine 104 (2024): 105163. 38772281 10.1016/j.ebiom.2024.105163 PMC11134892

[70] T. W. Powers , B. A. Neely , Y. Shao , et al., “MALDI Imaging Mass Spectrometry Profiling of N‐Glycans in Formalin‐Fixed Paraffin Embedded Clinical Tissue Blocks and Tissue Microarrays,” PLoS One 9, no. 9 (2014): e106255. 25184632 10.1371/journal.pone.0106255 PMC4153616

[71] R. R. Drake , T. W. Powers , K. Norris‐Caneda , A. S. Mehta , and P. M. Angel , “In Situ Imaging of N‐Glycans by MALDI Imaging Mass Spectrometry of Fresh or Formalin‐Fixed Paraffin‐Embedded Tissue,” Current Protocols in Protein Science 94, no. 1 (2018): e68. 30074304 10.1002/cpps.68

[72] E. N. Wallace , C. A. West , C. T. McDowell , et al., “An N‐Glycome Tissue Atlas of 15 Human Normal and Cancer Tissue Types Determined by MALDI‐Imaging Mass Spectrometry,” Scientific Reports 14, no. 1 (2024): 489. 38177192 10.1038/s41598-023-50957-w PMC10766640

[73] C. A. West , H. Liang , R. R. Drake , and A. S. Mehta , “New Enzymatic Approach to Distinguish Fucosylation Isomers of N‐Linked Glycans in Tissues Using MALDI Imaging Mass Spectrometry,” Journal of Proteome Research 19, no. 8 (2020): 2989–2996. 32441096 10.1021/acs.jproteome.0c00024 PMC8908332

[74] M. Orozco‐Moreno , E. A. Visser , K. Hodgson , et al., “Targeting Aberrant Sialylation and Fucosylation in Prostate Cancer Cells Using Potent Metabolic Inhibitors,” Glycobiology 33, no. 12 (2023): 1155–1171. 37847613 10.1093/glycob/cwad085 PMC10876042

[75] Galaxy C. The Galaxy Platform for Accessible, Reproducible and Collaborative Biomedical Analyses: 2022 Update,” Nucleic Acids Research 50, no. W1 (2022): W345–W351. 35446428 10.1093/nar/gkac247 PMC9252830

[76] M. Martin , Cutadapt Removes Adapter Sequences from High‐Throughput Sequencing Reads, vol. 17 (EMBnet, 2011), 3–10.

[77] D. Kim , B. Langmead , and S. L. Salzberg , “HISAT: A Fast Spliced Aligner With Low Memory Requirements,” Nature Methods 12, no. 4 (2015): 357–360. 25751142 10.1038/nmeth.3317 PMC4655817

[78] Y. Liao , G. K. Smyth , and W. Shi , “featureCounts: An Efficient General Purpose Program for Assigning Sequence Reads to Genomic Features,” Bioinformatics 30, no. 7 (2013): 923–930. 24227677 10.1093/bioinformatics/btt656

[79] C. W. Law , Y. Chen , W. Shi , and G. K. Smyth , “Voom: Precision Weights Unlock Linear Model Analysis Tools for RNA‐Seq Read Counts,” Genome Biology 15, no. 2 (2014): R29. 24485249 10.1186/gb-2014-15-2-r29 PMC4053721

[80] P. M. Valero‐Mora , “ggplot2: Elegant Graphics for Data Analysis,” Journal of Statistical Software Book Reviews 35, no. 1 (2010): 1–3.21603108

[81] M. D. Young , M. J. Wakefield , G. K. Smyth , and A. Oshlack , “Gene Ontology Analysis for RNA‐Seq: Accounting for Selection Bias,” Genome Biology 11, no. 2 (2010): R14. 20132535 10.1186/gb-2010-11-2-r14 PMC2872874

[82] M. Alhamdoosh , M. Ng , N. J. Wilson , et al., “Combining Multiple Tools Outperforms Individual Methods in Gene Set Enrichment Analyses,” Bioinformatics 33, no. 3 (2017): 414–424. 27694195 10.1093/bioinformatics/btw623 PMC5408797

[83] G. R. Warnes , B. Bolker , L. Bonebakker , et al., gplots: Various R Programming Tools for Plotting Data. R Package Version, vol. 3 (R package version, 2015).

[84] N. Hoti , S. Yang , Y. Hu , P. Shah , M. C. Haffner , and H. Zhang , “Overexpression of Alpha (1,6) Fucosyltransferase in the Development of Castration‐Resistant Prostate Cancer Cells,” Prostate Cancer and Prostatic Diseases 21, no. 1 (2018): 137–146. 29339807 10.1038/s41391-017-0016-7 PMC5895601

[85] N. Hoti , T. S. Lih , J. Pan , et al., “A Comprehensive Analysis of FUT8 Overexpressing Prostate Cancer Cells Reveals the Role of EGFR in Castration Resistance,” Cancers (Basel) 12, no. 2 (2020): 468. 32085441 10.3390/cancers12020468 PMC7072180

[86] X. Sun , D. Mahajan , B. Chen , Z. Song , and L. Lu , “A Quantitative Study of the Golgi Retention of Glycosyltransferases,” Journal of Cell Science 134, no. 20 (2021): 258564. 10.1242/jcs.258564 34533190

[87] S. F. Lichtenthaler , M. K. Lemberg , and R. Fluhrer , “Proteolytic Ectodomain Shedding of Membrane Proteins in Mammals‐Hardware, Concepts, and Recent Developments,” EMBO Journal 37, no. 15 (2018): e99456. 29976761 10.15252/embj.201899456 PMC6068445

[88] T. Hirata , M. Takata , Y. Tokoro , M. Nakano , and Y. Kizuka , “Shedding of N‐Acetylglucosaminyltransferase‐V Is Regulated by Maturity of Cellular N‐Glycan,” Communications Biology 5, no. 1 (2022): 743. 35915223 10.1038/s42003-022-03697-y PMC9343384

[89] N. C. Hait , A. Maiti , R. Wu , et al., “Extracellular Sialyltransferase st6gal1 in Breast Tumor Cell Growth and Invasiveness,” Cancer Gene Therapy 29, no. 11 (2022): 1662–1675. 35676533 10.1038/s41417-022-00485-y PMC9663294

[90] X. Wang , J. Chen , Q. K. Li , et al., “Overexpression of Alpha (1,6) Fucosyltransferase Associated With Aggressive Prostate Cancer,” Glycobiology 24, no. 10 (2014): 935–944. 24906821 10.1093/glycob/cwu051 PMC4153758

[91] C. Mao , J. Li , L. Feng , and W. Gao , “Beyond Antibody Fucosylation: Alpha‐(1,6)‐fucosyltransferase (Fut8) as a Potential New Therapeutic Target for Cancer Immunotherapy,” Antimicrobial Therapy 6, no. 2 (2023): 87–96. 10.1093/abt/tbad004 PMC10108557 37077473

[92] K. Yamasaki , T. Yamasaki , and H. Tateno , “The Trimeric Solution Structure and Fucose‐Binding Mechanism of the Core Fucosylation‐Specific Lectin PhoSL,” Scientific Reports 8, no. 1 (2018): 7740. 29773815 10.1038/s41598-018-25630-2 PMC5958098

[93] C. T. McDowell , X. Lu , A. S. Mehta , P. M. Angel , and R. R. Drake , “Applications and Continued Evolution of Glycan Imaging Mass Spectrometry,” Mass Spectrometry Reviews 42, no. 2 (2023): 674–705. 34392557 10.1002/mas.21725 PMC8946722

[94] H. Miyake , M. Pollak , and M. E. Gleave , “Castration‐Induced Up‐Regulation of Insulin‐Like Growth Factor Binding Protein‐5 Potentiates Insulin‐Like Growth Factor‐I Activity and Accelerates Progression to Androgen Independence in Prostate Cancer Models,” Cancer Research 60, no. 11 (2000): 3058–3064. 10850457

[95] D. Wang , C. Cheng , X. Chen , et al., “IL‐1beta Is an Androgen‐Responsive Target in Macrophages for Immunotherapy of Prostate Cancer,” Advanced Science (Weinheim) 10, no. 17 (2023): 2206889. 10.1002/advs.202206889 PMC10265092 37092583

[96] H. Li , J. E. Melnyk , B. X. H. Fu , et al., “Abstract B066: Genome‐Wide CRISPR Screens Identify PTGES3 as a Druggable AR Modulator,” Cancer Research 83, no. 11_Supplement (2023): B066.

[97] J. Li , A. D. Guillebon , J. W. Hsu , et al., “Human fucosyltransferase 6 enables prostate cancer metastasis to bone,” British Journal of Cancer 109, no. 12 (2013): 3014–3022. 24178760 10.1038/bjc.2013.690 PMC3859952

[98] N. M. Okeley , S. C. Alley , M. E. Anderson , et al., “Development of Orally Active Inhibitors of Protein and Cellular Fucosylation,” Proceedings of the National Academy of Sciences of the United States of America 110, no. 14 (2013): 5404–5409. 23493549 10.1073/pnas.1222263110 PMC3619284

[99] Y. Zhou , T. Fukuda , Q. Hang , et al., “Inhibition of Fucosylation by 2‐Fluorofucose Suppresses Human Liver Cancer HepG2 Cell Proliferation and Migration as Well as Tumor Formation,” Scientific Reports 7, no. 1 (2017): 11563. 28912543 10.1038/s41598-017-11911-9 PMC5599613

[100] M. A. Carrascal , M. Silva , J. S. Ramalho , et al., “Inhibition of Fucosylation in Human Invasive Ductal Carcinoma Reduces E‐Selectin Ligand Expression, Cell Proliferation, and ERK1/2 and p38 MAPK Activation,” Molecular Oncology 12, no. 5 (2018): 579–593. 29215790 10.1002/1878-0261.12163 PMC5928367

[101] N. M. Okeley , R. A. Heiser , W. Zeng , et al., “Abstract 5551: SGN‐2FF: A Small‐Molecule Inhibitor of Fucosylation Modulates Immune Cell Activity in Preclinical Models and Demonstrates Pharmacodynamic Activity in Early Phase 1 Analysis,” Cancer Research 78, no. 13_Supplement (2018): 5551.

[102] M. L. Disis , L. R. Corulli , E. A. Gad , et al., “Therapeutic and Prophylactic Antitumor Activity of an Oral Inhibitor of Fucosylation in Spontaneous Mammary Cancers,” Molecular Cancer Therapeutics 19, no. 5 (2020): 1102–1109. 32165557 10.1158/1535-7163.MCT-19-0500

[103] H. Tateno , S. Nakamura‐Tsuruta , and J. Hirabayashi , “Comparative Analysis of Core‐Fucose‐Binding Lectins From Lens Culinaris and *Pisum sativum* Using Frontal Affinity Chromatography,” Glycobiology 19, no. 5 (2009): 527–536. 19218400 10.1093/glycob/cwp016

[104] K. T. Do , L. Q. M. Chow , K. Reckamp , et al., “First‐In‐Human, First‐In‐Class, Phase I Trial of the Fucosylation Inhibitor SGN‐2FF in Patients With Advanced Solid Tumors,” Oncologist 26, no. 11 (2021): 925–e1918. 34288257 10.1002/onco.13911 PMC8571760

[105] J. F. A. Pijnenborg , E. A. Visser , M. Noga , et al., “Cellular Fucosylation Inhibitors Based on Fluorinated Fucose‐1‐Phosphates*,” Chemistry 27, no. 12 (2021): 4022–4027. 33336886 10.1002/chem.202005359 PMC7986151

[106] J. F. A. Pijnenborg , E. Rossing , J. Merx , et al., “Fluorinated Rhamnosides Inhibit Cellular Fucosylation,” Nature Communications 12, no. 1 (2021): 7024. 10.1038/s41467-021-27355-9 PMC8640046 34857733

[107] Y. Lv , Z. Zhang , S. Tian , W. Wang , and H. Li , “Therapeutic Potential of Fucosyltransferases in Cancer and Recent Development of Targeted Inhibitors,” Drug Discovery Today 28, no. 1 (2023): 103394. 36223858 10.1016/j.drudis.2022.103394

[108] N. M. Okeley , R. A. Heiser , W. Zeng , et al., “SGN‐2FF: A Small‐Molecule Inhibitor of Fucosylation Modulates Immune Cell Activity in Preclinical Models and Demonstrates Pharmacodynamic Activity in Early Phase 1 Analysis,” Cancer Research 78, no. 13_Supplement (2018): 5551.

[109] E. Rossing , J. F. A. Pijnenborg , and T. J. Boltje , “Chemical Tools to Track and Perturb the Expression of Sialic Acid and Fucose Monosaccharides,” Chemical Communications (Cambridge, England) 58, no. 87 (2022): 12139–12150. 36222364 10.1039/d2cc04275d PMC9623448

[110] M. Wang , Z. Zhang , M. Chen , et al., “FDW028, a Novel FUT8 Inhibitor, Impels Lysosomal Proteolysis of B7‐H3 via Chaperone‐Mediated Autophagy Pathway and Exhibits Potent Efficacy Against Metastatic Colorectal Cancer,” Cell Death & Disease 14, no. 8 (2023): 495. 37537172 10.1038/s41419-023-06027-0 PMC10400579

[111] Y. Lv , Z. Zhang , M. Wang , et al., “Discovery of Novel FUT8 Inhibitors With Promising Affinity and In Vivo Efficacy for Colorectal Cancer Therapy,” Bioorganic Chemistry 149 (2024): 107492. 38820939 10.1016/j.bioorg.2024.107492

[112] Y. Manabe , T. Takebe , S. Kasahara , et al., “Development of a FUT8 Inhibitor With Cellular Inhibitory Properties,” Angewandte Chemie (International Ed. in English) 63, no. 52 (2024): e202414682. 39340265 10.1002/anie.202414682

[113] P. A. Gilormini , V. N. Thota , A. Fers‐Lidou , et al., “A Metabolic Inhibitor Blocks Cellular Fucosylation and Enables Production of Afucosylated Antibodies,” Proceedings of the National Academy of Sciences of the United States of America 121, no. 27 (2024): e2314026121. 38917011 10.1073/pnas.2314026121 PMC11228515

[114] X. Wang , J. Gu , E. Miyoshi , K. Honke , and N. Taniguchi , “Phenotype Changes of Fut8 Knockout Mouse: Core Fucosylation Is Crucial for the Function of Growth Factor Receptor(s),” Methods in Enzymology 417 (2006): 11–22. 17132494 10.1016/S0076-6879(06)17002-0

[115] H. Guo and K. L. Abbott , “Functional Impact of Tumor‐Specific N‐Linked Glycan Changes in Breast and Ovarian Cancers,” Advances in Cancer Research 126 (2015): 281–303. 25727151 10.1016/bs.acr.2014.11.006

[116] Y. C. Liu , H. Y. Yen , C. Y. Chen , et al., “Sialylation and Fucosylation of Epidermal Growth Factor Receptor Suppress Its Dimerization and Activation in Lung Cancer Cells,” Proceedings of the National Academy of Sciences of the United States of America 108, no. 28 (2011): 11332–11337. 21709263 10.1073/pnas.1107385108 PMC3136320

[117] P. Hu , B. Shi , F. Geng , C. Zhang , W. Wu , and X. Z. Wu , “E‐Cadherin Core Fucosylation Regulates Nuclear Beta‐Catenin Accumulation in Lung Cancer Cells,” Glycoconjugate Journal 25, no. 9 (2008): 843–850. 18553167 10.1007/s10719-008-9144-6

[118] M. Okada , S. Chikuma , T. Kondo , et al., “Blockage of Core Fucosylation Reduces Cell‐Surface Expression of PD‐1 and Promotes Anti‐Tumor Immune Responses of T Cells,” Cell Reports 20, no. 5 (2017): 1017–1028. 28768188 10.1016/j.celrep.2017.07.027

[119] C. J. Logothetis and S. H. Lin , “Osteoblasts in Prostate Cancer Metastasis to Bone,” Nature Reviews. Cancer 5, no. 1 (2005): 21–28. 15630412 10.1038/nrc1528

[120] N. Zhang , M. Li , X. Xu , et al., “Loss of Core Fucosylation Enhances the Anticancer Activity of Cytotoxic T Lymphocytes by Increasing PD‐1 Degradation,” European Journal of Immunology 50, no. 11 (2020): 1820–1833. 32460355 10.1002/eji.202048543

