# Supplementary material for: FUT8 Is a Critical Driver of Prostate Tumour Growth and Can Be Targeted Using Fucosylation Inhibitors
Source: Cancer Med. 2025 May 19;14(10):e70959. doi: 10.1002/cam4.70959 (PMC12086987; doi:10.1002/cam4.70959)

Supplementary Figure 1

FUT8 is upregulated in high grade prostate tumours and repressed by androgens in prostate cancer cells

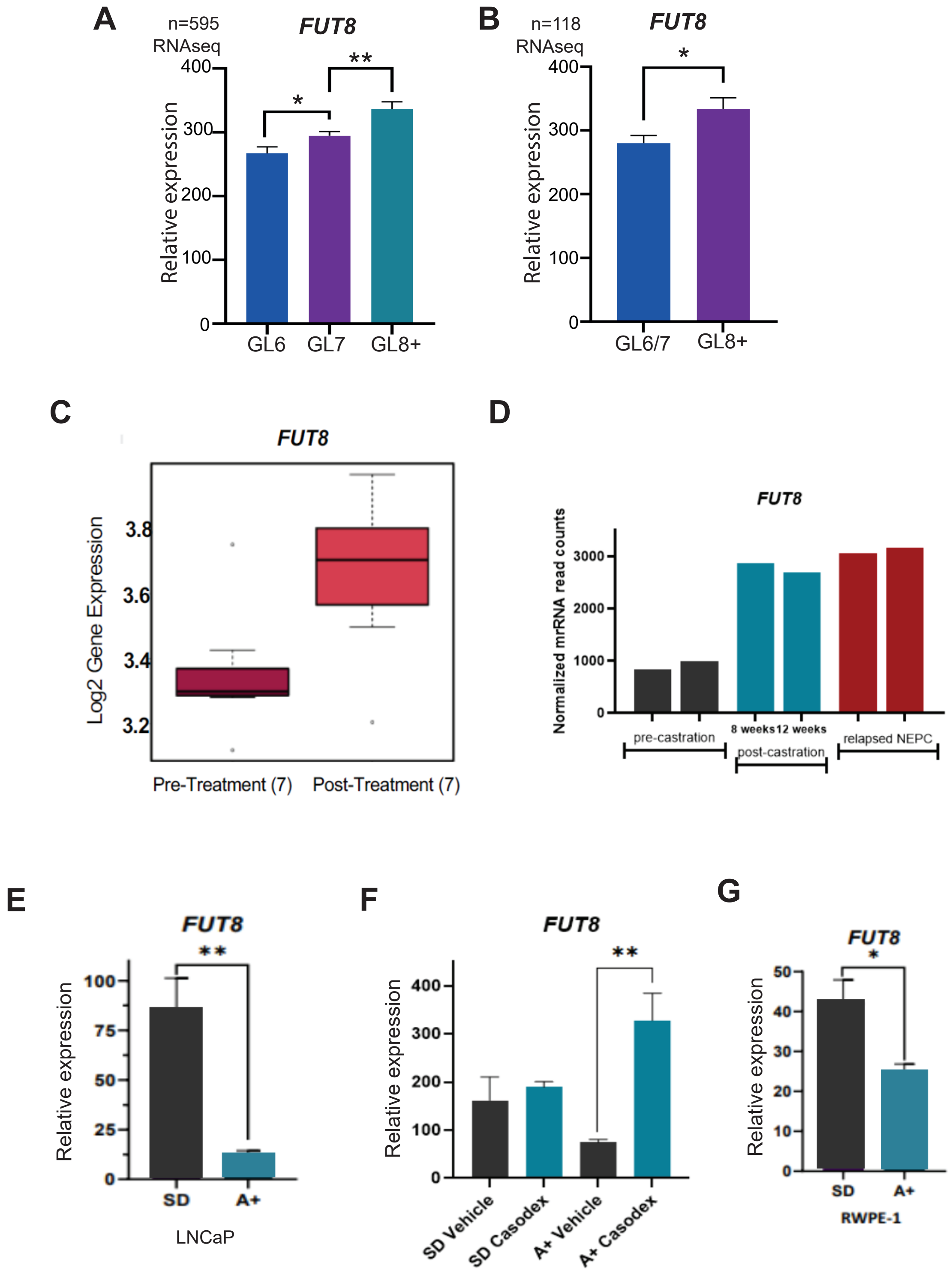

Supplementary Figure 2
Validation of FUT8 antibody for immunohistochemistry

A. Immunohistochemistry staining of FFPE PC3 cell pellets depleted of FUT8 using shRNA

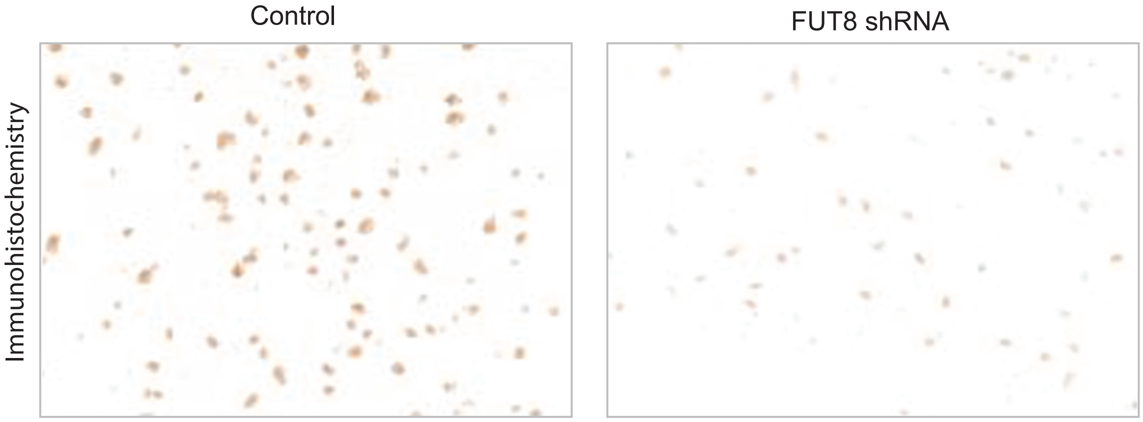

Supplementary Figure 3
Validation of prostate cancer cells with knockdown or overexpression of FUT8

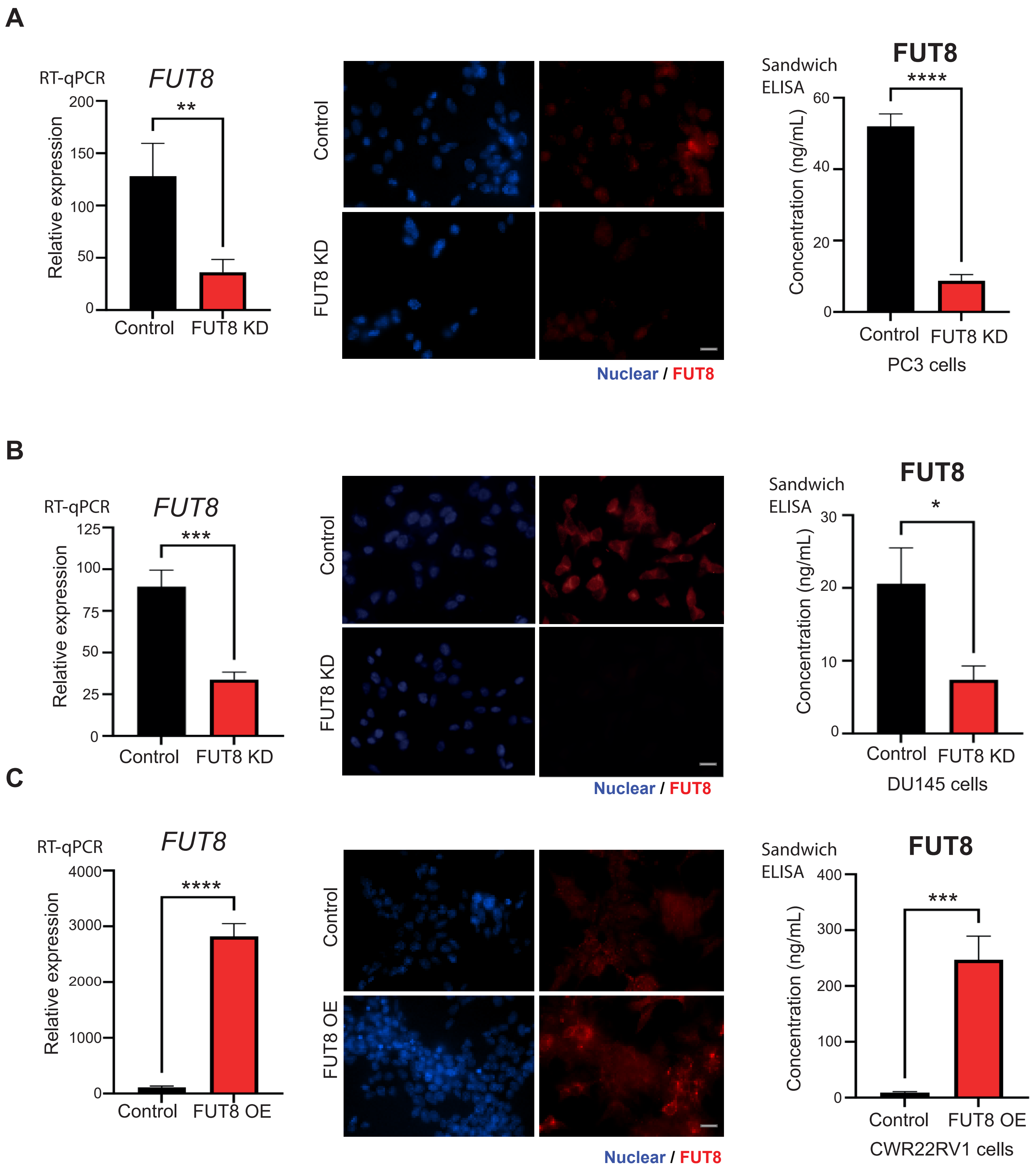

Supplementary Figure 4

FUT8 promotes prostate cancer cell growth and colony formation

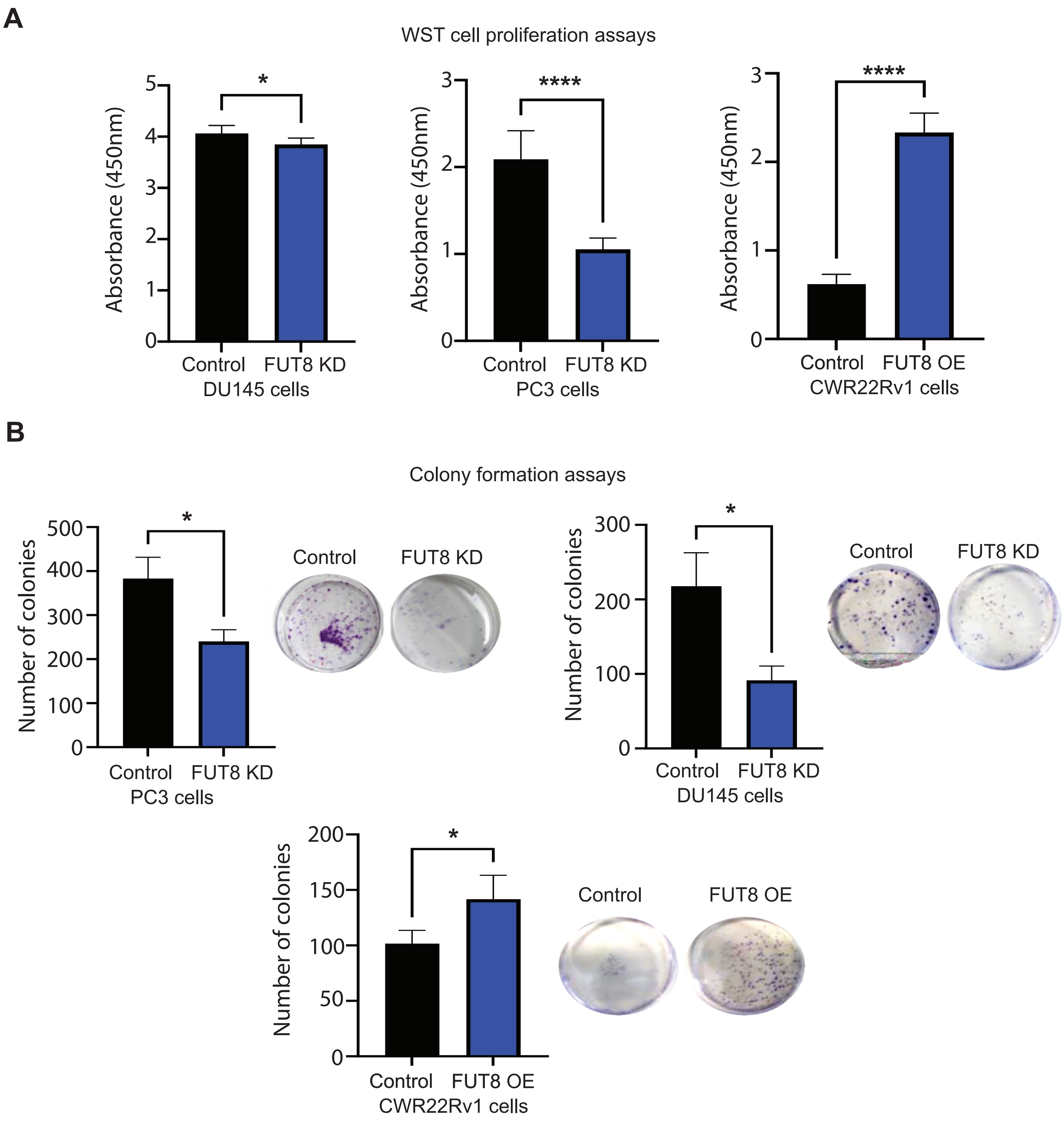

Supplementary Figure 5
Knockdown of FUT8 in DU145 prostate cancer cells reduces core fucosylation (monitored using PhoSL immunofluorescence)

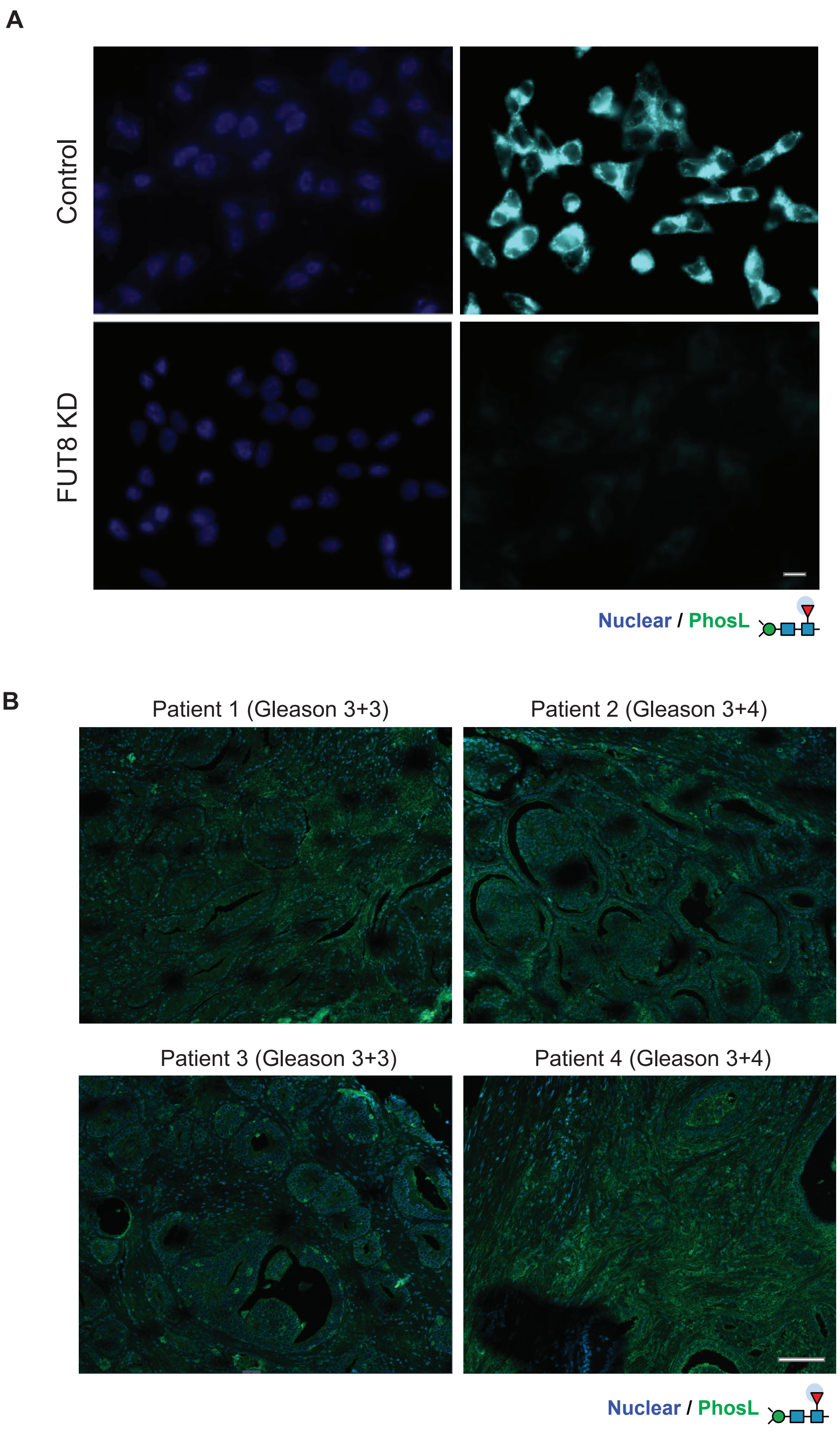

Supplementary Figure 6
RNAseq analysis of PC3 cells with knockdown of FUT8

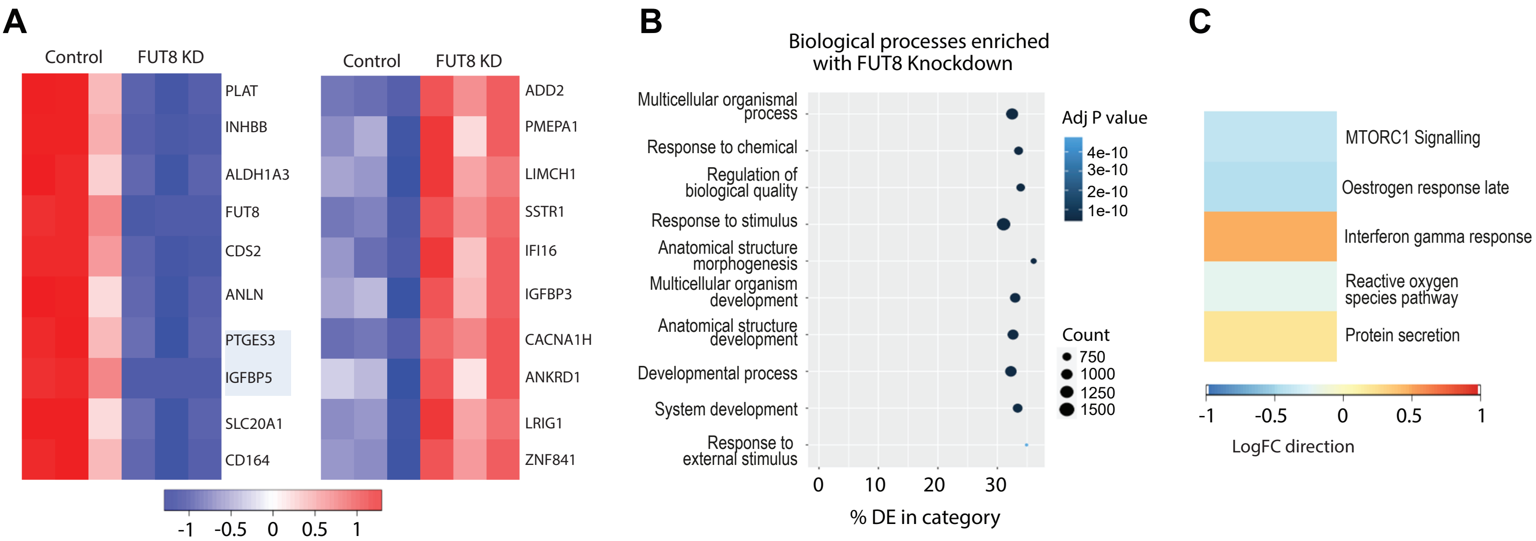

Supplementary Figure 7
Fucosylation inhibitors suppress the growth of prostate cancer cells

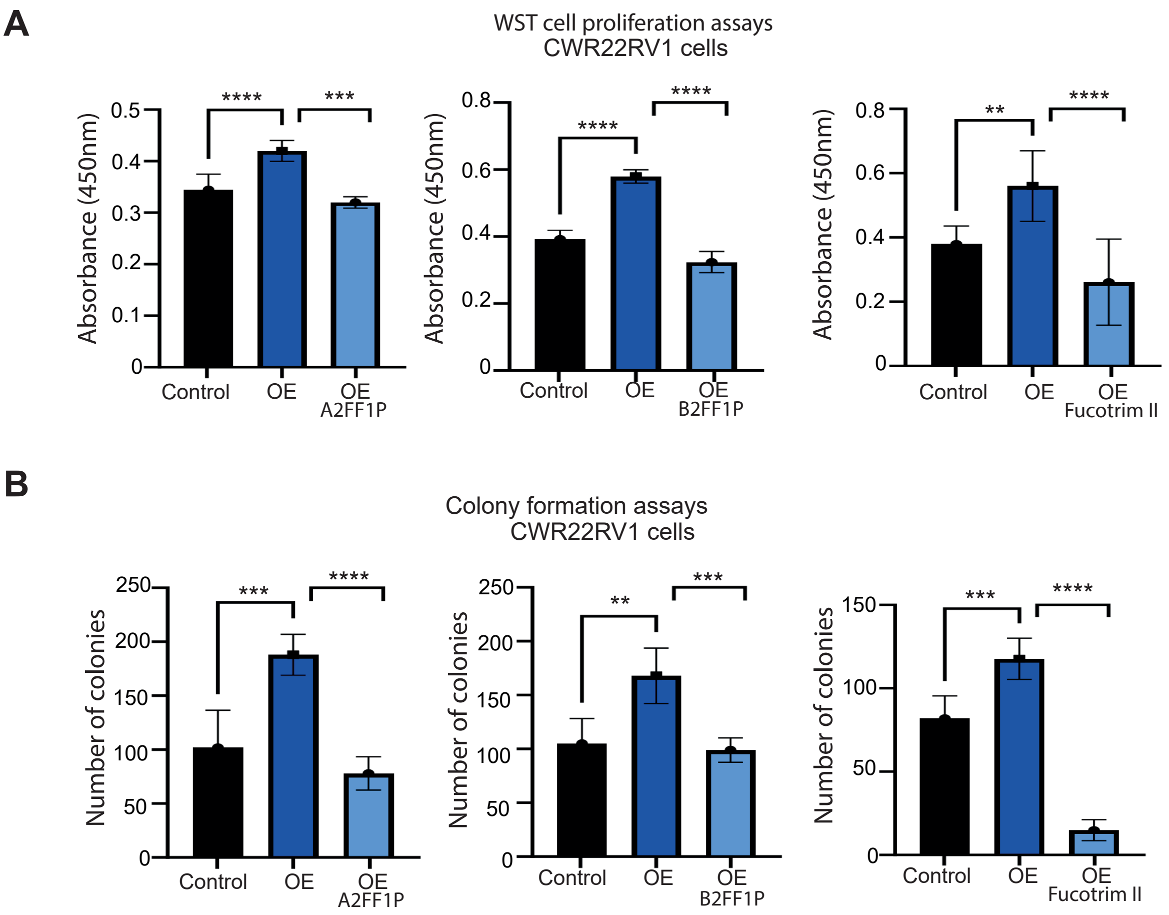

Supplementary Figure 8
Fucotrim I blocks fucose incorporation in TRAMPC2 and RM1 prostate cancer cells

A

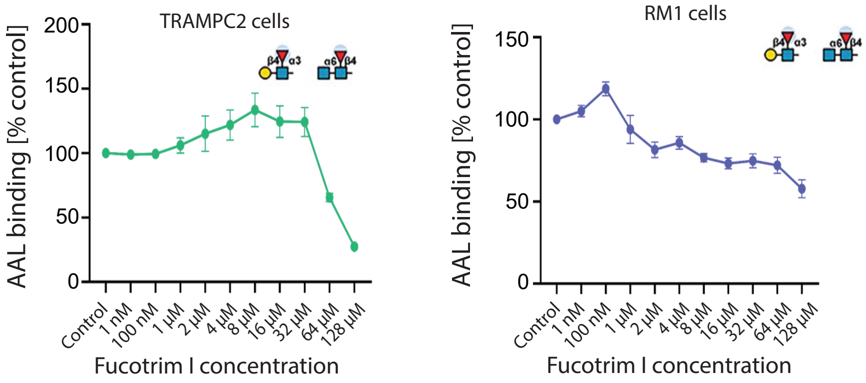

Supplement: Supplementary file 1 — Figure S1. Figure S2. Figure S3. Figure S4. Figure S5. Figure S6. Figure S7. Figure S8. [file CAM4-14-e70959-s001.pdf]
